# Effectiveness of Gamification in Knee Replacement Rehabilitation: Protocol for a Randomized Controlled Trial With a Qualitative Approach

**DOI:** 10.2196/38434

**Published:** 2022-11-28

**Authors:** Eeva Aartolahti, Maarit Janhunen, Niina Katajapuu, Juha Paloneva, Konsta Pamilo, Airi Oksanen, Hannes Keemu, Mikko Karvonen, Mika Luimula, Raija Korpelainen, Timo Jämsä, Keijo Mäkelä, Ari Heinonen

**Affiliations:** 1 Institute of Rehabilitation JAMK University of Applied Sciences Jyväskylä Finland; 2 Faculty of Sport and Health Sciences University of Jyväskylä Jyväskylä Finland; 3 Faculty of Health and Well-being Turku University of Applied Sciences Turku Finland; 4 Department of Surgery Central Finland Healthcare District and University of Eastern Finland Jyväskylä Finland; 5 Department of Orthopedics Coxa Hospital for Joint Replacement Tampere Finland; 6 Department of Orthopedics and Traumatology Turku University Hospital and University of Turku Turku Finland; 7 Faculty of Business and Engineering Turku University of Applied Sciences Turku Finland; 8 Department of Sports and Exercise Medicine Oulu Deaconess Institute Foundation sr Oulu Finland; 9 Research Unit of Population Health University of Oulu Oulu Finland; 10 Medical Research Center Oulu Oulu University Hospital and University of Oulu Oulu Finland; 11 Research Unit of Medical Imaging, Physics and Technology University of Oulu Oulu Finland

**Keywords:** knee arthroplasty, serious game, gamification, therapeutic exercise, rehabilitation, physical therapy, Kinect, mixed methods, randomized controlled trial

## Abstract

**Background:**

Exergames can provide encouraging exercise options. Currently, there is limited evidence regarding home-based exergaming in the postoperative phase of total knee replacement (TKR).

**Objective:**

This study aimed to investigate the effects of a 4-month postoperative home-based exergame intervention with an 8-month follow-up on physical function and symptoms among older persons undergoing TKR compared with home exercise using a standard protocol. In addition, a concurrent embedded design of a mixed methods study was used by including a qualitative component within a quantitative study of exergame effects.

**Methods:**

This was a dual-center, nonblinded, two-arm, parallel group randomized controlled trial with an embedded qualitative approach. This study aimed to recruit 100 patients who underwent their first unilateral TKR (aged 60-75 years). Participants were randomized to the exergame or standard home exercise arms. Participants followed a custom-made exergame program independently at their homes daily for 4 months. The primary outcomes at 4 months were function and pain related to the knee using the Oxford Knee Score questionnaire and mobility using the Timed Up and Go test. Other outcomes, in addition to physical function, symptoms, and disability, were game user experience, exercise adherence, physical activity, and satisfaction with the operated knee. Assessments were performed at the preoperative baseline and at 2, 4, and 12 months postoperatively. Exergame adherence was followed from game computers and using a structured diary. Self-reported standard exercise was followed for 4 months of intervention and physical activity was followed for 12 months using a structured diary. Qualitative data on patients’ perspectives on rehabilitation and exergames were collected through laddering interviews at 4 and 12 months.

**Results:**

This study was funded in 2018. Data collection began in 2019 and was completed in January 2022. The COVID-19 pandemic caused an unavoidable situation in the study for recruitment, data collection, and statistical analysis. As of November 2020, a total of 52 participants had been enrolled in the study. Primary results are expected to be published by the end of 2022.

**Conclusions:**

Our study provides new knowledge on the effects of postoperative exergame intervention among older patients with TKR. In addition, this study provides a new understanding of gamified postoperative rehabilitation, home exercise adherence, physical function, and physical activity among older adults undergoing TKR.

**Trial Registration:**

ClinicalTrials.gov NCT03717727; https://clinicaltrials.gov/ct2/show/NCT03717727

**International Registered Report Identifier (IRRID):**

RR1-10.2196/38434

## Introduction

### Background and Rationale

People with osteoarthritis (OA) often remain sedentary after total knee replacement (TKR) [1,2], predisposing them to noncommunicable diseases and disabilities with advancing age. Exercise is a recommended core treatment in the clinical guidelines for musculoskeletal disorders and a cornerstone of standard care for patients with OA [3,4]. People with knee OA should follow similar doses of exercise (frequency, intensity, time, type, volume, and progression) as physical activity (PA) recommended by public health authorities to have a positive effect on physical function [5]. Although engagement in PA is difficult for the general population, it is even more challenging for people with OA. They are often physically inactive and highly sedentary because of pain and disease progression [6].

Typically, TKR surgery is performed after years of progressive pain, swelling, stiffness, and decreased range of motion in the joint. The rate of TKRs for severe OA is increasing substantially [7,8], and it is one of the most frequently performed elective surgeries [9]. Fast tracking for TKR involves optimizing the treatment protocol to reduce the primary treatment period and thus reduce the use of institutional care [10]. The primary treatment period after TKR was approximately 3 days, and for 75% of the patients, the discharge destination was home [10]. Emphasis is placed on providing postoperative physiotherapy in hospitals to enable the patient’s independence in activities of daily living (ADL) before discharge. In addition, hospital physiotherapists supervise active home exercises to improve knee range of motion, muscle strength, and weight-bearing while walking. High-intensity outpatient physical therapy directly after discharge improves the functional performance of patients who had undergone TKR [11]. Nonetheless, rehabilitation after discharge is mainly the patient’s responsibility.

A study by Valtonen et al [12] found that although the operation reduces pain effectively, even 10 months after the knee replacement surgery, the knee extension torque and power in the operated lower limb remained weaker than those in the nonoperated side. This asymmetry limits patients’ ability to negotiate stairs [12]. Questionnaires administered to patients 5 years after TKR have shown that their expectations regarding postoperative PA were not fulfilled to the same extent as their expectations regarding pain relief [13]. PA levels remain below the recommended levels, and sedentary behavior continues after TKR [1]. However, meeting preoperative expectations is a significant determinant of overall patient satisfaction following lower limb joint arthroplasty [14].

In the long term, PA would be crucial for the aging population undergoing TKR by reducing the risk of chronic noncommunicable diseases and disabilities in ADL. Exercise adherence is an essential predictor of the long-term effectiveness of exercise therapy both within and after the intervention period [15]. Gamified exercise, or exergaming, is a new approach to increase exercise adherence. Increased exercise adherence using exergames has been reported in several studies involving different patient groups [16,17].

### Objectives

This study explored the effectiveness of home-based exergame rehabilitation on physical function and symptoms after TKR. This “Gamification in Knee Replacement Rehabilitation, randomized controlled trial” (BEE-RCT) is part of the “Business Ecosystems in Effective Exergaming” (BEE) project. The primary objective of this study was to examine the effectiveness of a 4-month gamified home exercise program on physical function, disability, and symptoms following TKR surgery. We hypothesized that people assigned to the exergame arm would have better functional status and lower levels of symptoms than those assigned to the standard home exercise arm.

Furthermore, we will assess exercise adherence and game experience in the gamified home-based rehabilitation arm of the study following TKR surgery and the maintenance of intervention benefits 1 year after TKR surgery. In addition, using a mixed methods approach, we aim to investigate the complex interrelationship between gamified rehabilitation, exercise adherence, PA, physical function, and symptoms in older adults after TKR surgery.

## Methods

### Trial Design

**Figure 1:**
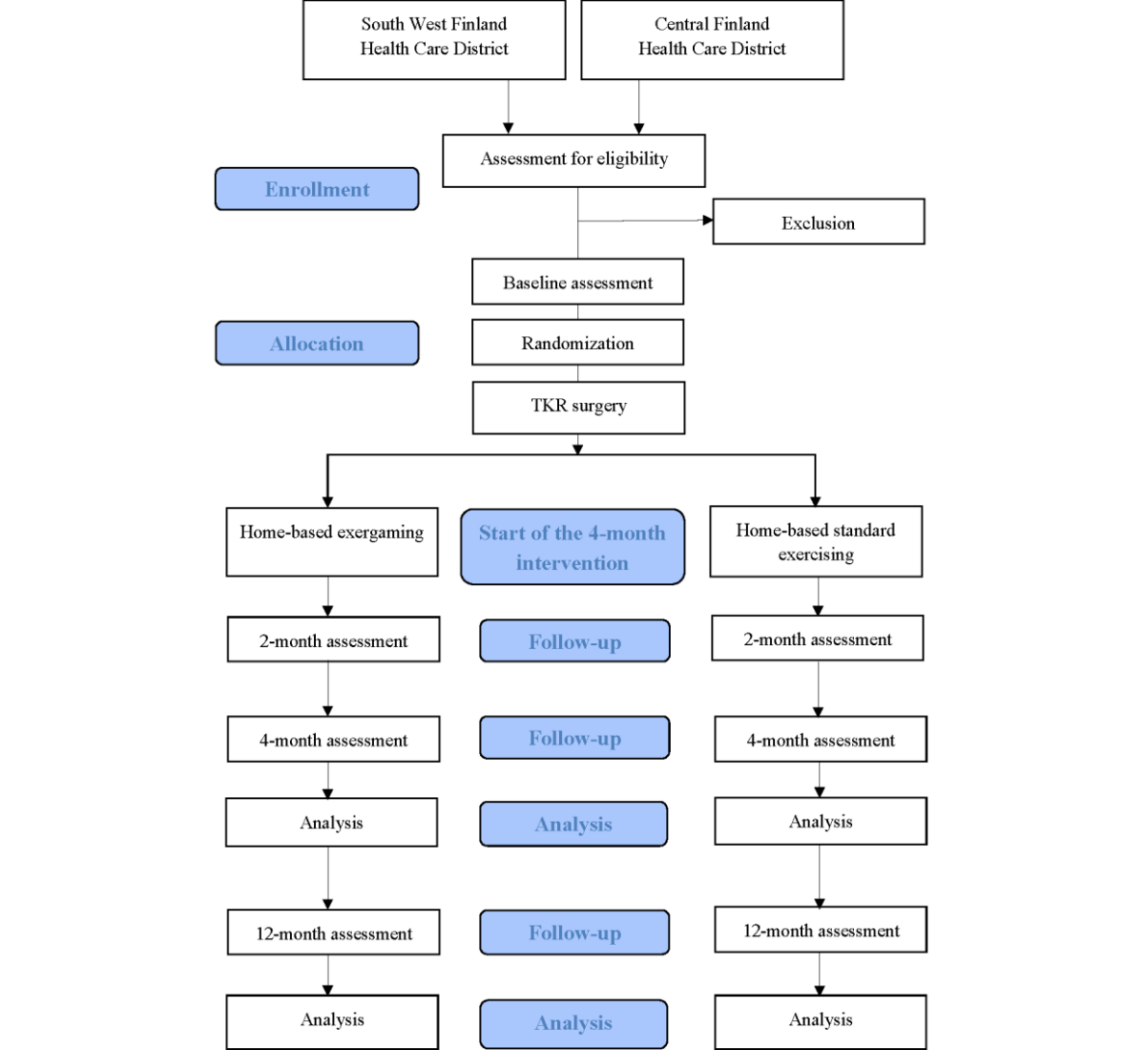
Flowchart of the Gamification in Knee Replacement Rehabilitation, randomized controlled trial. TKR: total knee replacement.

This was a 4-month dual-center, two-arm, parallel group randomized controlled trial that determined the effectiveness of exergame-based home-based rehabilitation by comparing it with standard home exercise after TKR. The 4-month intervention period was followed by the 8-month follow-up period. Participants’ perspectives on rehabilitation and exergames were explored using an embedded qualitative approach. Figure 1 illustrates the flow of this study.

**Figure 2:**
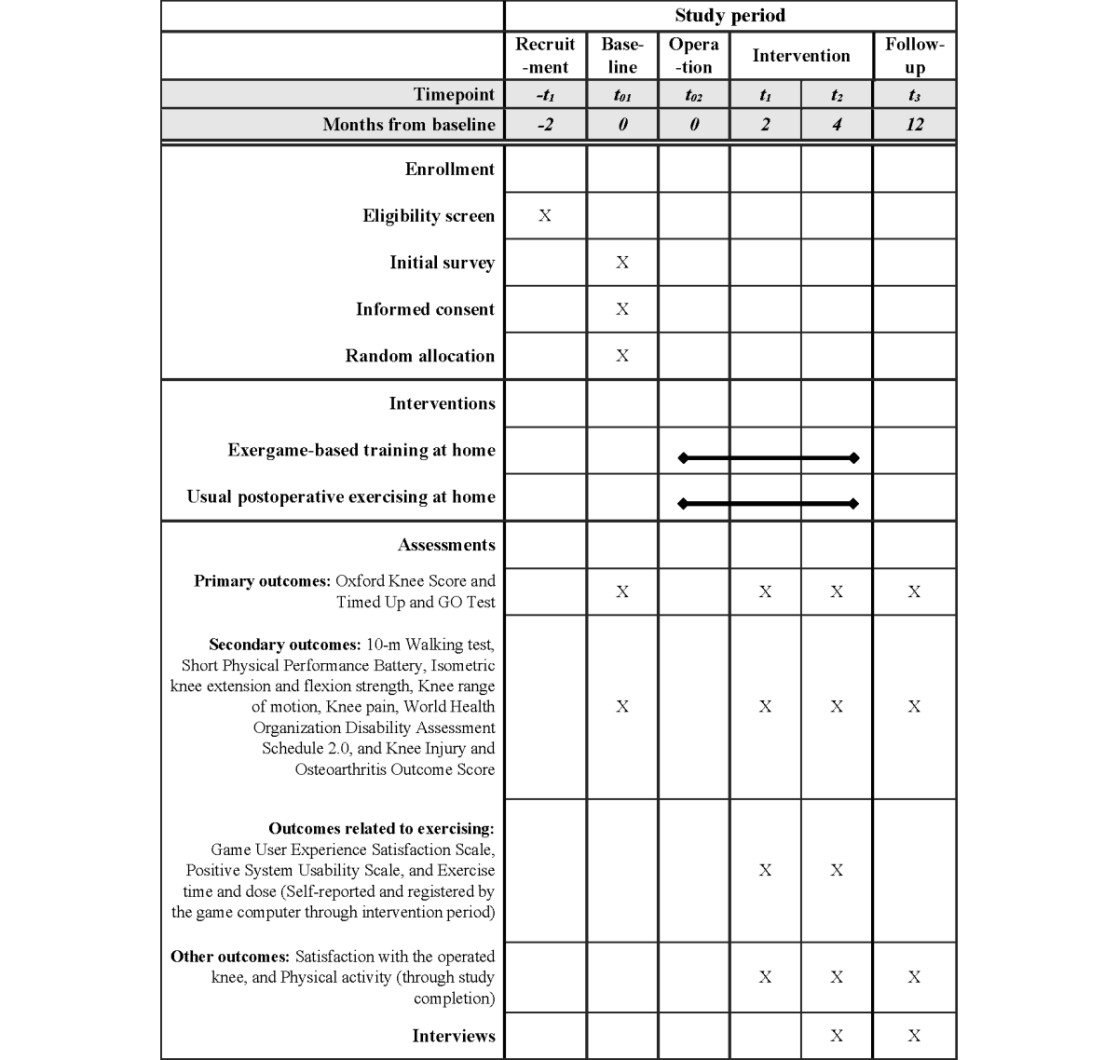
The schedule of enrollment, interventions, assessments, and interviews in the Gamification in Knee Replacement Rehabilitation, randomized controlled trial.

Figure 2 presents the enrollment schedule, interventions, outcome assessments, and interviews. The participants’ functioning, disability, and symptoms were assessed preoperatively *at t_0_* (baseline) and postoperatively *at t_1_*, *t_2_*, *and t_3_* (2nd, 4th, and 12th month, respectively). Individually arranged assessments were conducted in the exercise laboratory at the Turku University of Applied Sciences and the exercise laboratory at the University of Jyväskylä. Research data were collected before TKR surgery at baseline and during the RCT intervention and follow-up periods. The baseline assessment (*t_0_*) was performed within 2 weeks before the planned day of surgery. During the baseline assessment visit, participants provided written informed consent, after which the research physiotherapist measured the outcomes, performed randomization, and guided home exercising according to the allocation group. The postoperative assessments (*t_1_,* *t_2_*, and *t_3_*) were performed within +5 or −5 days of the postoperative 2-, 4-, and 12-month time points. During the postoperative assessment visit, the research physiotherapist measured the outcomes (*t_1,_* *t_2_*, and *t_3_*) and guided the PA of standard care and the gym work out for strength training (*t_2_)*.

### Study Setting

The study participants were Finnish patients with knee OA who lived in the Southwest and Central Finland Health Care Districts and had undergone TKR surgery at the Turku University Hospital in the City of Turku or the Central Finland Hospital in the City of Jyväskylä. These hospitals perform approximately 1700 (Turku, 1200 and Jyväskylä, 500) knee joint arthroplasties per year. After discharge from the hospital, patients performed therapeutic exercises independently in their homes for 4 months with either exergame (intervention group) or standard postoperative exercises (control group). The intervention group participants followed the standard postoperative home exercise program when they could not use exergames, such as during vacations in another locality or abroad. For 1 year after surgery, the participants maintained a record of PA.

### Eligibility Criteria

The study participants were men and women aged 60-75 years who had undergone TKR surgery. They were recruited during their preoperative outpatient visits at the Turku University Hospital and Central Finland Hospital. Inclusion criteria for enrollment in the study were as follows: (1) aged 60 to 75 years; (2) first primary, unilateral TKR; (3) diagnoses of primary knee arthrosis (International Classification of Diseases 10th Revision codes M17.0 and M17.1); (4) mechanical axis of the limb in varus; (5) model of the TKR is posterior stabilizing or cruciate-retaining prosthesis; and (6) normal vision with or without eyeglasses. The exclusion criteria were as follows: (1) diagnosed memory disorder or cognitive impairment; (2) neurological conditions (such as Parkinson disease, multiple sclerosis, or stroke); and (3) fractures, rheumatoid arthritis, or other biomechanical disruptions in the affected lower limb within 1 year before surgery. Inclusion criterion for age was set to have a sample presenting a vast majority of patients who had undergone TKR without trauma background or increased number of comorbidities and to control high variability in skills of technology use.

### Recruitment

During the preoperative outpatient hospital visit, the surgeon, with either a physiotherapist or a research nurse, assessed the eligibility of study patients. If a patient was eligible, a physiotherapist or research nurse described the study to the patient and asked for written permission for the research’s initial preoperative contact. The patient was informed of the study course during the initial phone contact. The date of the preoperative baseline assessment was agreed upon by interested patients who did not report conditions that could affect the safety of physical testing and exercise. These conditions included chest pain during exercise or other physical exertions, excessive shortness of breath, cardiac medication, seizures or unconsciousness, fainting, or dizziness. In the baseline assessment, eligibility was reassessed based on the initial survey. The patient provided written informed consent to participate in the study if eligible.

### Random Allocation

After the preoperative baseline measurements, the study participants were randomly allocated one by one to the exergame and standard home exercise groups. A person unrelated to the study had previously used a computer-generated randomization procedure using blocks of 2 and 4 in random order and according to the place of recruitment (Jyväskylä or Turku), gender and baseline Timed Up and Go (TUG) test with time ≤10 seconds (indicating faster performance in patients who had undergone TKR [18,19]). A person unrelated to the study had concealed group allocation cards in opaque sealed envelopes into 4 separate sets of stratification (slow men, slow women, fast men, and fast women) per place of recruitment. After the baseline measurement of each participant, the research physiotherapist of the exercise laboratory opened the next envelope from the appropriate set of envelopes, complying with the allocation stratification, and assigned the participant to the exergame or standard home exercise group according to the group allocation card in the envelope.

### Interventions

#### Exergame

**Figure 3:**
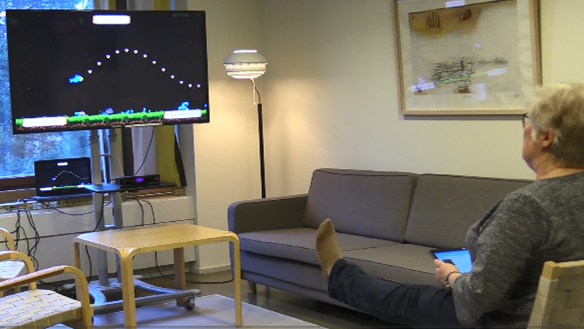
Exergame solution in rehabilitation after total knee replacement in the home setting.

The exergame group played 11 different Unity (Unity Technologies) exergames (Turku University of Applied Sciences, Finland) developed in the BEE project. Exergame development was based on a standard postoperative rehabilitation program for TKR. The exergames were played using a motion sensor (Kinect 2.0, Microsoft Corporation) connected to a laptop (Micro-Star International) and controlled with a tablet (Lenovo). Training software (GoodLife Kiosk Trainer, GoodLife Technology) and television screens offered implementation in the homes of the study participants (Figure 3). Exergaming content and progression were built for players using PhysioTools Online (PhysioTools) exercise library software, enhanced with exergames. The player controls the games using movements similar to the standard postoperative home exercise program. The research physiotherapist instructed the participants in the exergame group to play exergames progressively several times a day for 16 weeks after discharge from the hospital (Multimedia Appendix 1). In addition, the participants were recommended to follow the standard postoperative home exercise program when they could not use exergames, such as during vacations in another locality or abroad.

The initial guidance for setting up the exergame system, using applications, and testing 2 games (the Bubble Runner and Cave Game) took place at the exercise laboratory baseline visit immediately after randomization. Face-to-face individual guidance was provided for 15 to 20 minutes by the same research physiotherapist who had conducted the baseline assessment. In addition, the study participants received a written manual on exergame equipment, setup, applications, and exergaming. The participants independently set up the exergame equipment in their homes.

Exergames were categorized according to exercise target: knee extension and flexion, knee flexion or squatting, weight shifting from side to side, stretching, and functional exergames (Multimedia Appendix 1). The knee extension-flexion games (the Cave Game and Intruders) and stretching game (the Cannon) were played in a sitting position, whereas the other games (the Rowing Game, Pick Up, Squat Pong, Bubble Runner, Hat Trick, Brick Breaker, Hiking, and Toy Golf) were played in a standing position. Detailed information on each exergame’s solution explaining the game’s course and the strategies and metrics used is provided in Multimedia Appendix 1.

The first week of the exergame protocol (Multimedia Appendix 1) was initiated on the third day after surgery. The exergames of each week were prescribed to be played in the order presented in Multimedia Appendix 1. The protocol specified 4 to 5 exergames for each intervention week. The participants were instructed to play the games prescribed several times a day. Weekly, the exergame protocol became more demanding as games became more challenging, and playing time, number of repetitions, sets, and intensity in games increased. In weeks 5-12, exergames that had once been in the exergame protocol but were not planned for the current week were further available for play as additional exergames of the week. In the last 4 weeks of the exergame protocol (weeks 13-16), the exergames were the most demanding versions, with the highest exercising doses.

#### Control

The control group underwent a standard postoperative home exercise program initially instructed by a physiotherapist in the hospital where the surgery was performed (Multimedia Appendices 1-3). The research physiotherapist instructed the control group participants to follow this standard program several times a day for 16 weeks starting after discharge from the hospital. Similar to the exergame group, guidance was provided at the exercise laboratory baseline visit immediately after randomization. Face-to-face individual guidance was provided for 5 to 10 minutes by the same research physiotherapist who had conducted the baseline assessment.

#### Postoperative Usual Care

Both groups received standard care after TKR (Multimedia Appendices 2 and 3). Standard care included a clinical visit to hospital physiotherapists or orthopedists at an average of 2 months after surgery. In addition, the artificial joint status and ability to function were monitored regularly with electronic surveys and, if necessary, conventional radiography. Surveys were taken 2 to 3 months after surgery, 1 year after surgery, and will be taken every 5 years thereafter. In addition, PA guidance based on PA recommendations [20] was also provided as a part of standard care. At the 4-month postoperative research assessment visit, the research physiotherapist briefly, in approximately 5 minutes, counseled the participants of both groups about the PA guidance of standard care and provided written gym instructions for strength training (Multimedia Appendix 1).

### Outcome Measures

#### Overview

Research physiotherapists responsible for collecting descriptive data and outcomes pre- and postoperatively were trained. Preoperative descriptive data were gathered by an initial survey, by interviewing study participants, and from medical records with the permission of the study participants. The selection of outcomes (Figure 2) adapted the recommended tests of physical function in people with knee OA [21] and were collected during exercise laboratory visits in Turku or Jyväskylä, according to the participant’s residential area. Before data collection, participants were asked about their current perceived well-being (normal, tired, or unwell) to ensure safe exercise laboratory measurements. The outcomes were collected in a permanent order during both pre- and postoperative visits. The questionnaires were collected first, followed by height and weight measurements to calculate BMI. The questionnaires were collected using the pen and paper method in the following order: Oxford Knee Score (OKS), Knee injury and Osteoarthritis Outcome Score (KOOS), World Health Organization Disability Assessment Schedule (WHODAS 2.0), and satisfaction with the operated knee. In addition, on the 2- and 4-month visits, after previous questionnaires, the Positive System Usability Scale (P-SUS) and Game User Experience Satisfaction Scale (GUESS) questionnaires were collected from participants in the intervention group. Subsequently, the assessments were performed for all participants in the following order: knee pain (visual analog scale [VAS]), knee range of motion, Short Physical Performance Battery (SPPB), TUG test, 10-meter walking speed, and isometric knee extension and flexion strength. Sitting balance was measured using a prototype measuring device (SmartChair, Tohoku University) in the TUG and SPPB sit-to-stand tests. Finally, interviews were conducted. Data regarding exercise time and dose throughout the intervention period and PA through study completion were gathered by self-reported structured diaries and exergame time and dose data by game computers. The outcome assessments and interviews are described in detail in the *Primary Outcomes*, *Secondary Outcomes*, and *Qualitative Data* sections.

To minimize the amount of missing data owing to the COVID-19 pandemic, the principal investigators approved changes to the data collection method. Consequently, all data to be collected using the pen and paper method were collected by mail from the participants during the lockdowns and from the participants who, on their own, did not visit the exercise laboratory after the lockdown. The change to the data collection method did not require a modification of the ethical statement or hospital permission.

#### Primary Outcomes

Changes in knee function and pain were assessed using the OKS questionnaire [22]. OKS is a short, reproducible, valid, and sensitive questionnaire used to assess clinically important changes after TKR surgery [23]. Each of the 12 items was scored from 0 to 4, with 4 being the best outcome. When summed, the overall scores ranged from 0 to 48 points, with higher scores indicating better function and lower knee pain.

The TUG test was used to assess changes in mobility [24]. The TUG test was performed twice at maximum speed, with the shoes on. Time in seconds was measured using a stopwatch. Both results were recorded, and a better result (shorter time in seconds) was used. A lower TUG test time indicated better mobility.

#### Secondary Outcomes

Walking was assessed with a 10-meter walking test [25] performed 4 times with shoes on: twice with normal speed and twice with fast speed with a 2-meter flying start. Possible use of walking aids was also recorded. The time in seconds was measured using photocells. All results in seconds were recorded and reported as the walking speed (m/s). Better results at normal and fast speeds (higher value of m/s) were used. Higher speed in the 10-meter walking test indicated better walking performance.

Lower extremity performance was assessed using the SPPB test [26], which includes 3 subtests: static balance in 3 different positions, 4-meter walking time, and 5 repetitions of sit-to-stand. The balance test was performed without shoes. The walking test was performed at normal speed, and the sit-to-stand test was performed at fast speed, and both these tests were performed with shoes on. The possible use of a walking aid during the test was also recorded. All subtest results were recorded in seconds and scored from 0 to 4 points, leading to a maximum of 12 points. Higher SPPB test scores indicate better lower extremity performance.

Muscle strength was assessed with isometric knee extension and flexion forces collected using a Metitur Good Strength dynamometer in Jyväskylä (Newton [N]) and a Con-Trex Multijoint dynamometer (Newton-meter [Nm]) in Turku. Knee extension and flexion forces were measured from each lower limb in the sitting position, with the knee angle at 60° of flexion. The measurement sequence was as follows: (1) knee extension, (2) knee flexion force from the nonoperated lower limb, (3) knee extension, and (4) knee flexion force from the operated lower limb. The participants performed 4 warm-up and training contractions with moderate force, after which 4 contractions with maximal force were measured. Thereafter, the performances were repeated until the force improved to <50 N during the knee extension contraction and <20 N during the knee flexion contraction. One contraction lasted approximately 3 seconds, and there was a 30-second break between contractions. All maximal force results were recorded. In addition, after completion of the sets of contractions of the lower limb (eg, knee extensions and flexions of the operated lower limb), the participants were asked if the pain impaired their performance. The direction of painful performance (ie, extension, flexion, or both) and pain intensity on a scale of 1 to 10 were recorded. The best results for knee extension and flexion forces (Nm or N) were used. For analysis, N values were calculated as Nm with the formula Force (N) × lever arm length of the leg (m) and normalized based on body weight (Nm/weight). A higher value in the isometric knee extension and flexion tests indicated better muscle strength.

The range of knee joint flexion and extension was measured using a universal goniometer [27,28]. Measurements were performed on the operated knee in the supine position. First, active and passive knee flexion were measured, followed by active and passive knee extensions. The results were recorded in degrees; a smaller result in extension and larger result in flexion indicated a better joint range of motion.

Knee pain was assessed using the pen and paper version of the VAS [29] by asking participants to rate the average knee pain over a week. The VAS ranges from 0 to 100, indicating no knee pain and the worst possible knee pain, respectively.

Disability was evaluated using the self-reported WHODAS 2.0 questionnaire [30] including 12 items. The scale ranges from 0 to 4 per item, indicating values from “no difficulty” to “extreme difficulty,” and the total score ranges from 0 to 48 points. A lower total score on the WHODAS 2.0 indicated better function.

The KOOS questionnaire [31] includes 5 subscales: pain, other symptoms, quality of life, ADL, and sport and recreation function. The 5-point Likert scale ranged from 0 to 4 for each subscale. The maximum score on each subscale was 100 points. A higher score on the KOOS questionnaire indicated less knee pain and associated problems.

Exergame experience was evaluated using the GUESS [32] and the 10-item P-SUS [33] questionnaires. GUESS includes 9 subscales that assess usability or playability, narratives, play engrossment, enjoyment, creative freedom, audio esthetics, personal gratification, social connectivity, and visual esthetics. The questions were scored from 1 to 7, indicating values from “strongly disagree” to “strongly agree.” In addition, a score of 8 indicates “not applicable.” P-SUS questions were scored from 1 to 5, indicating values from “strongly disagree to “strongly agree.” A higher score on the GUESS and P-SUS questionnaires indicated a more positive experience of the exergames played.

Outcomes related to exercise adherence were collected using a self-reported structured diary and a game computer. Participants filled in the exercise diary daily for the exergame and standard home exercise adherence (number and duration of exercise sessions in minutes). This will be summed as days, sets, and minutes of exergaming and standard exercising per week. Minutes per week will be calculated as metabolic equivalent hours per week [34-36]. In addition, exergame adherence was measured based on the game computer data gathered during each exergame session (name of exergame, duration, timestamp, and game score). Adherence from game computer will be calculated as days, sets, and minutes of performed exergaming per week. Longer exercise duration, that is, minutes per week, indicated a higher level of commitment to exercise. In addition, adherence throughout the intervention period will be reported as the percentage of completed exercise days compared with the total number of days.

PA was measured using a structured PA diary, which the participants completed daily from baseline to 12 months. The diary form sought data on each activity’s mode, intensity, and duration. Leisure time PA and PA related to daily errands or commuting are reported separately. PA will be calculated as the metabolic equivalent hours per week and month by multiplying each marked activity by self-evaluated intensity (1, light; 2, moderate; and 3, vigorous), according to Ainsworth et al [34].

Satisfaction with the operated knee was assessed using the following question: “How satisfied are you with your operated knee?” Response options were “very satisfied,” “satisfied,” “dissatisfied,” and “very dissatisfied.”

#### Qualitative Data

Individual interview data on patients’ perspectives on rehabilitation and exergames were collected 4 and 12 months after the operation. Face-to-face interviews were recorded, and laddering notations were used. Owing to the COVID-19 pandemic, phone interviews were conducted as necessary. All participants were invited to participate in the interviews. The laddering interview technique supports the understanding of the user’s perspective [37]. The technique is based on the personal construct theory [38], which emulates human beings’ mental models. The laddering interviewing technique operationalizes the personal construct theory by providing a means to investigate system attributes, consequences (reasoning) for system use, and values or goals that drive its use [37]. The laddering interview process by Tuunanen and Peffers [39] was followed. First, participants were given a list of stimuli (Multimedia Appendix 4) intended to suggest ideas about possible recovery experiences of participants to enable brainstorming [37]. Second, the interviewees were asked to select 2 stimuli that they perceived as the most important to them. The interviewer proceeded to ask the participant to explain why each experience was important by asking questions such as “why” and “why that would be important.” The ladder data were recorded as attribute-consequence-value chains, as presented by Tuunanen and Peffers [39]. The interview recordings are also transcribed to later enable other qualitative approaches in the analysis.

### Adverse Events and Dropouts

Adverse events that the study participants reported spontaneously were recorded. The type of harm and its onset time, intensity, and frequency were recorded, and the possible causal relationship with the intervention was assessed. The date, informant, and reason for dropout were recorded for the study participants who discontinued the study. In addition, we assessed whether the interruption was because of an adverse event, the participant’s state of health, personal will, or whether the participant was not reached.

### Blinding (Masking)

Participants and trial personnel were not blinded after the point of assignment to interventions following baseline measurements because of the nature of the interventions and outcomes assessed. Participants knew which group they belonged to because the group-specific exercise routines were followed immediately after randomization, and gaming equipment was provided to those randomized to the intervention group. Trial personnel, in turn, were able to deduce the group from the questionnaires related to the game experience gathered during 2- and 4-month visits from participants randomized to the exergame group.

### Promotion of Participant Retention

Participant retention was promoted with phone contact before the 2-, 4-, and 12-month measurements to ensure participation. Data collection was supported by mailing questionnaires to participants when they did not come to the exercise laboratory for 2-, 4-, and 12-month measurements owing to personal reasons or pandemic constraints. The outcomes collected by mail were OKS, VAS, WHODAS 2.0, KOOS, satisfaction with the operated knee, GUESS, and P-SUS scores.

### Data Management

The management of the collected and generated data was documented in the Data Management Plan (DMP) document. The data were pseudonymized and stored securely in locked cupboards or protected network drives. Unidentifiable data were shared with the investigators involved in the study. Processes to promote data quality include the documented data collection protocol and training of data handlers. All research results will be made public through conference presentations or journal publications. Individual study participants will not be identifiable based on public research results. A data monitoring committee was not needed because of the short duration of the trial and the known minimal risks.

### Sample Size

The intended sample size (n=100) of the BEE-RCT was based on the primary outcome, the OKS [22]. Assuming a mean difference of 5 (SD 8) points [40] in a change in the OKS between the groups at 6 to 12 months [40,41], a sample size of 42 in the intervention and control groups was required to detect differences between groups at an α of .05 and reach power of 80%. The dropout rate was estimated to be 10%; consequently, a minimum of 100 participants were needed to be recruited. In 2020, the COVID-19 pandemic decreased the number of elective surgeries, such as TKR, and there were lockdowns in exercise laboratories. Due to this, recruitment slowed in the spring of 2020 and was completely stopped for 3 months because of lockdowns. Consequently, the trial failed to recruit the intended sample size of participants.

### Data Analysis Plan

Repeated measures of the changes in primary and secondary outcomes will be compared between the exergame and standard home exercise groups using intention-to-treat analysis, mixed effects models, and an unstructured covariance structure. Fixed effects included group, time, and group × time interactions. In the primary analysis, repeated measurements will be taken at different time points, including at baseline and at 2 and 4 months. Mixed models allow for the analysis of unbalanced data sets without imputation; therefore, all available data with the full analysis set will be analyzed. In response to the impact of COVID-19, the number of participants is expected to be insufficient for per-protocol analysis. The maintenance of intervention benefits for 12 months will be analyzed using similar mixed effects models as secondary analyses.

A concurrent embedded design of a mixed methods study was used by including a qualitative component within a quantitative study of exercise effectiveness. In the mixed methods approach, quantitative data analysis is mainly descriptive to support the interpretation of qualitative findings. For physical function measures, categorization based on a clinically meaningful level or change from baseline will be used to further integrate the qualitative data. With the qualitative interview data, primarily thematic coding and clustering of the concepts arising from ladder chains are planned [37]. The ladder data will be coded and categorized by 2 researchers and checked until a complete consensus is achieved between them. Integration of quantitative and qualitative data will focus on developing a framework using the identified clusters and structuring them by adopting the interpretative structure modeling technique [42].

### Ethics and Dissemination

#### Ethics Approval

The Ethics Committee of the Southwest Finland Health Care Districts has reviewed and made a statement for the BEE-RCT study (Dnro 66/1801/2018, June 19, 2018) according to the Medical Research Decree. In addition, the Turku University Hospital and Central Finland Central Hospital provided permission for the study.

This study follows the guidelines of the Finnish National Board on Research Integrity [43], the ethical principles of the University of Jyväskylä, and good scientific practice and valid legislation. The ethical issues of this study concern humans as participants, the use and storage of the data, and principles related to intellectual property rights.

We conducted a pilot study with laboratory loading measurements before RCT to ensure safe and reasonable exergaming for patients recovering from TKR [44]. The universities insured the participants of the duration of laboratory measurements and trips directly related to them.

#### Consent or Assent

This project involved human participants, and informed consent was obtained along with the BEE-RCT. The research physiotherapist obtained consent before the baseline measurements. Consent follows the ethical principles of research in the human, social, and behavioral sciences and data protection legislation. It was ensured that the potential participants had fully understood the information and did not feel pressured or coerced to provide consent. Participants could deny or cancel their participation at any time without any consequences. The patient’s consent or refusal to participate did not affect the treatment they received.

#### Confidentiality

All personal and collected data were treated as confidential at all stages of the study and were stored separately. All personal data were stored in a highly secure Deltagon CollabRoom environment. The electronic data were saved with metadata in university network drives, which are protected by usernames and passwords. The participant ID list that connects the study participants and research data will be disposed of after 15 years. Institutions hold the ownership of registry data. Data collected in this research will be owned by the University of Jyväskylä, the Turku University of Applied Sciences, and the University of Oulu. The intellectual property rights policy of each university will be followed, and ownership has been agreed upon when creating the DMP.

#### Declaration of Interests

This project is part of the BEE collaboration project and is partly funded by companies. The data will be processed and published without any commercial interest or input. Funders did not interpret the results of this project or participate in writing and publishing the research results. The BEE publication committee supervises and conforms to the authorships and contributions to each planned publication. The principal investigator will own all the results concerning collaborations with commercial entities. Commercial collaborations already own the rights to their products, and this will not be disputed.

#### Access to Data

Researchers from the University of Jyväskylä, the Turku University of Applied Sciences, and the University of Oulu will have access to the final data. The right to access data is managed according to the joint controller agreement of universities.

#### Dissemination Policy

Metadata entries will be published via the University of Jyväskylä Converis research information system. Individual written feedback was provided to the participants from participant-level data. The feedback included individual results of primary and secondary outcomes measured at baseline and after the intervention and follow-up periods. The trial results will be reported via scientific publications and congress presentations. In publications, the recommendations of International Committee of Medical Journal Editors for authorship eligibility will be followed [45].

The DMP for this project supports the reuse of data. However, as the data consist of confidential personal information, their use is strictly restricted to research purposes explicitly mentioned in the project plan. The project metadata will be found via the Converis research information system. Metadata, complete with a full description of methods, will be published as data sets are saved in the Jyväskylä University Digital Repository. Data are available to external collaborators upon agreement on the terms of data use and publication of results.

#### Protocol Version and Amendments

This study refers to protocol version 3 on March 16, 2020. Before data collection, PA diaries replaced activity monitors to measure PA. Important modifications in response to extenuating circumstances, such as COVID-19, have been reported following the CONSERVE-SPIRIT extension for reporting trial protocols and completed trials [46] (Multimedia Appendices 5 and 6).

## Results

The first participant was recruited in November 2018 and randomly allocated to the intervention or control group in March 2019. The intervention was initiated in March 2019. Owing to the COVID-19 pandemic, trial recruitment was paused from March 16, 2020, to May 31, 2020. Recruitment was completed in December 2020, and 52 participants provided informed consent. Of these participants, 88% (46/52) completed at least one postoperative measurement (*t_1_*, *t_2_*, and *t_3_*). The trial ended in January 2022, when the last recruited participant completed the 12-month follow-up measures and interviews (*t_3_*). Primary results are expected to be published by the end of 2022.

## Discussion

### Principal Findings

The randomized controlled multicenter trial BEE-RCT will provide new knowledge about the gamification of therapeutic exercise and changes in physical function, disability, and symptoms after TKR surgery. First, we hypothesize that patients with TKR who were assigned to the exergame group would demonstrate better functioning and lower level of symptoms when compared with those assigned to the standard home exercise group. Second, we hypothesize that exercise adherence would be higher in the exergame group and that there would be an association between adherence and positive user and game experience. Using a mixed methods approach, we expect to better understand the complex interrelationship between gamified rehabilitation, exercise adherence, PA, physical function, and symptoms in older adults after TKR surgery.

In therapeutic exercises, training adherence dictates the effectiveness of exercise interventions in real life. Traditionally, printed instructions have reminded patients who had undergone TKR to follow the recommended rehabilitation process at home. Knee replacement reduces pain effectively; nevertheless, engagement in exercise and PA is especially difficult for people with OA who remain sedentary after TKR [47,48]. In light of these results, new solutions for rehabilitation technology are welcome to support people in their behavior change process after surgery. More recently, remote technologies such as mobile apps, wearable sensors, and gamified solutions have been developed to assess, guide, motivate, and receive feedback during the rehabilitation process.

### Comparison With Prior Work

In BEE-RCT, we will explore the effects of custom-made exergames played in home settings. At the same time, previous studies of virtual reality tools after TKR have emphasized supervised interventions with standard commercial games [49] or without gamification [50]. Home-based rehabilitation will become even more essential after the COVID-19 pandemic, as the landscape of clinical outpatient rehabilitation changes from in-person visits to a greater reliance on remote services and digital rehabilitation [51].

Technological developments have enabled the digitalization of rehabilitation services. Traditionally, physiotherapists do not have valid and resource-effective methods to follow home exercise adherence or progression of patients after TKR. In BEE-RCT, the effects of postoperative home exercises and compliance will be studied thoroughly. The game computer gathered information on home exergame adherence, offering the advantage of objectively measuring which parts of the intervention participants were truly engaged in at home. This type of data provides the possibility of a large data set of exercise adherence, and it can be a valid measure of exercise behavior. In the future, new forms of data may be tested, and theories of intervention implementation and behavior change may be developed [52].

### Strengths and Limitations

The data collection for the BEE-RCT was implemented during the COVID-19 pandemic affecting people and society. Within the allotted time, fewer participants were recruited than was initially planned. Therefore, the study is likely not powered to reveal differences in primary outcomes between the exergame and usual home exercise groups. However, the strength of the BEE-RCT is the intervention planned for the home environment and continued without disruption during the pandemic. After the COVID-19 lockdown, few of the recruited participants considered that coming to the postoperative exercise laboratory assessments would pose a high risk of developing the disease, and therefore, they did not want to come for the scheduled visits. However, we collected all questionnaires, interviews, and intervention data. When interpreting the results, it should be considered that blinding participants and professionals did not exist except at baseline assessment before randomization.

In addition to effectiveness, any new technology must be feasible, appropriate, and meaningful before implementing evidence-based rehabilitation. By using a mixed methods approach and integrating quantitative and qualitative data, BEE-RCT will produce an in-depth understanding of older people’s experiences of their recovery process after TKR and the meanings of gamification. With a 1-year follow-up of physical function and PA combined with data from repeated interviews on patient experiences, we can explore complex interrelationships between gamified rehabilitation, exercise adherence, PA, physical function, and symptoms in older adults after TKR surgery.

### Conclusions

Building on a multidisciplinary approach, this project can generate new knowledge and relevant results for digital rehabilitation services and technology development. A dual-center randomized controlled trial will add data to the evidence regarding the effects of exergame interventions. A mixed methods design and novel laddering approach will generate data on the complex phenomena of PA and rehabilitation after TKR.

## Appendix

Multimedia Appendix 1 Exercise protocols in Gamification in Knee Replacement Rehabilitation, randomized controlled trial (BEE-RCT).

Multimedia Appendix 2 The guidebook of the Turku University Hospital for patients who had undergone knee replacement (in Finnish).

Multimedia Appendix 3 The guidebook of the Central Finland Hospital, Jyväskylä, for patients who had undergone knee replacement.

Multimedia Appendix 4 Laddering interview stimuli.

Multimedia Appendix 5 SPIRIT (Standard Protocol Items: Recommendations for Interventional Trials) checklist.

Multimedia Appendix 6 CONSERVE-SPIRIT Extension (CONSORT and SPIRIT extension for randomized controlled trials revised in extenuating circumstances).

## Abbreviations

ADL: activities of daily living
BEE: Business Ecosystems in Effective Exergaming project
BEE-RCT: Gamification in Knee Replacement Rehabilitation, randomized controlled trial
DMP: Data Management Plan
GUESS: Game User Experience Satisfaction Scale
KOOS: Knee injury and Osteoarthritis Outcome Score
OA: osteoarthritis
OKS: Oxford Knee Score
PA: physical activity
P-SUS: Positive System Usability Scale
SPPB: Short Physical Performance Battery
TKR: total knee replacement
TUG: Timed Up and Go
VAS: visual analog scale
WHODAS 2.0: World Health Organization Disability Assessment Schedule
