# Supplementary material for: Effectiveness of Gamification in Knee Replacement Rehabilitation: Protocol for a Randomized Controlled Trial With a Qualitative Approach
Source: JMIR Res Protoc. 2022 Nov 28;11(11):e38434. doi: 10.2196/38434 (PMC9745648; doi:10.2196/38434)
Supplement: Multimedia Appendix 1 [file resprot_v11i11e38434_app1.docx]

# Multimedia Appendix 1: Exercise protocols in BEE-RCT

1. Progressive 16-weeks exergame protocol in BEE-RCT
2. BEE-RCT exergames: Exercise targets, strategies, and metrics
3. Standard home exercise protocols for total knee replacement patients
4. Gym instructions were given to TKR patients at a four-month post-operative research assessment visit

**1.**

**Progressive 16-weeks exergame protocol in BEE-RCT**

|  | KNEE EXTENSION-FLEXION | | | | KNEE FLEXION/SQUATTING | | | | WEIGHT SHIFTING | | | STRETCHING | |  | FUNCTIONAL | | | |
| --- | --- | --- | --- | --- | --- | --- | --- | --- | --- | --- | --- | --- | --- | --- | --- | --- | --- | --- |
| Week | Game 1 | Reps | Time. *s* | Sets | Game 2 | Reps | Time, *s* | Sets | Game 3 | Time, *s* | Sets | Game 4 | Time, *s* | Sets | Game 5 | Time, *s* | Sets |  |
|  |  |  |  |  |  |  |  |  |  |  |  |  |  |  |  |  |  |  |
| 1 | Cave Game | 5 |  | 2 | Rowing Game (S) | 15 | 120 | 1 | Bubble Runner | 90 | 1 | Cannon | 120 | 1 |  |  |  |  |
| 2 | Cave Game | 7 |  | 2 | Rowing Game (M) | 17 | 120 | 1 | Bubble Runner | 120 | 2 | Cannon | 120 | 1 |  |  |  |  |
| 3 | Cave Game | 10 |  | 2 | Rowing Game (M) | 17 | 120 | 1 | Bubble Runner | 120 | 2 | Cannon | 120 | 1 |  |  |  |  |
| 4 | Cave Game | 10 |  | 3 | Rowing Game (F) | 25 | 120 | 1 | Hat Trick (S) | 120 | 2 | Cannon | 120 | 1 |  |  |  |  |
| 5 | Cave Game | 12 |  | 3 | Pick Up |  | 120 | 2 | Hat Trick (M) | 150 | 2 | Cannon | 120 | 1 |  |  |  |  |
| 6 | Intruders (S) | 10 | 120 | 2 | Pick Up |  | 150 | 2 | Hat Trick (M) | 180 | 2 | Cannon | 120 | 1 |  |  |  |  |
| 7 | Intruders (M) | 10 | 120 | 2 | Pick Up |  | 180 | 2 | Hat Trick (F) | 180 | 2 | Cannon | 120 | 1 | Hiking | 180 | 1 |  |
| 8 | Intruders (M) | 10 | 120 | 3 | Pick Up |  | 180 | 2 | Brick Breaker | 120 | 2 | Cannon | 120 | 1 | Hiking | 240 | 1 |  |
| 9 | Intruders (F) | 10 | 120 | 3 | Squat Pong (S) |  | 60 | 2 | Brick Breaker | 120 | 2 | Cannon | 120 | 1 | Toy Golf | 360 | 1 |  |
| 10 | Cave Game | 12 |  | 3 | Squat Pong (M) |  | 90 | 2 | Brick Breaker | 180 | 2 | Cannon | 120 | 1 | Toy Golf | 360 | 1 |  |
| 11 | Cave Game | 12 |  | 3 | Squat Pong (M) |  | 120 | 2 | Brick Breaker | 180 | 2 | Cannon | 120 | 1 | Toy Golf | 360 | 1 |  |
| 12 | Cave Game | 12 |  | 3 | Squat Pong (F) |  | 180 | 2 | Hat Trick (F) | 180 | 2 | Cannon | 120 | 1 | Toy Golf | 360 | 1 |  |
|  |  |  |  |  |  |  |  |  |  |  |  |  |  |  |  |  |  |  |
| 13-16 | Players choose at least one | | | | Players choose at least one | | | | Players choose at least one | | | Cannon | 120 | 1 | Players choose at least one | | | |
|  | Cave Game | 12 |  | 3 | Rowing Game (F) | 25 | 120 | 1 | Bubble Runner | 120 | 2 |  |  |  | Hiking | 240 | 1 |  |
|  | Intruders (F) | 10 | 120 | 3 | Pick Up |  | 180 | 2 | Brick Breaker | 180 | 2 |  |  |  | Toy Golf | 360 | 1 |  |
|  |  |  |  |  | Squat Pong (F) |  | 180 | 2 | Hat Trick (F) | 180 | 2 |  |  |  |  |  |  |  |
| (S) = Slow pace, (M) = Moderate pace, (F) = Fast pace | | | | | | | | | | | | | | | | | | |

| **2.**  **BEE-RCT exergames: Exercise targets, strategies, and metrics** The blue-white figure in the game image shows the posture in which the player is playing the exergame. | | | | |  |
| --- | --- | --- | --- | --- | --- |
| Exercise target | Exergame |  | Strategies | Metrics | |
|  |  |  |  |  | |
| Knee extension-flexion | 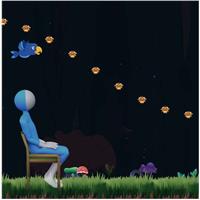 | CaveGame  The player collects as many bugs as possible by moving the Avatar (bird) upwards (knee extension) and downwards (knee flexion). | The algorithm sets repetitions and minimum degree values to knee extension-flexion movement. | Repetitions  Scope of movement | |
|  | 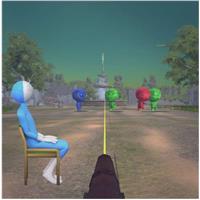 | Intruders  The player loads the cannon (knee flexion) and shoots (knee extension) zombies. The aiming of the cannon is done by hand movement. | The algorithm sets repetitions and minimum range of motion to knee extension-flexion movement based on players' functional performance. The knee extension-flexion movement must be performed in velocity set in the algorithm. | Repetitions  Scope of movement  Velocity (Slow → Moderate → Fast) | |
| Knee flexion/ Squat | 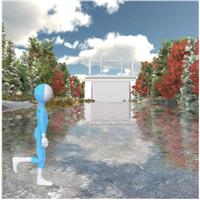 | RowingGame  The player rows the boat (knee flexion) with the aim to reach the gate before it closes. | The algorithm sets playing time and minimum range of motion to knee flexion movement. The flexion movement must be performed in the velocity set in the algorithm. | Time  Scope of movement  Velocity (Slow → Moderate → Fast) | |
|  | 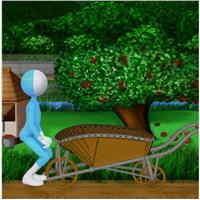 | PickUp  The player moves the Avatar (girl) in the garden so that it picks up vegetables (squat; down) and throws them to the wheelbarrow (squat; up). | The algorithm sets playing time and minimum range of motion to squat movement based on players' body dimensions. | Time  Scope of movement | |
|  | 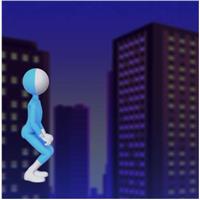 | SquatPong  The player plays tennis against the computer by moving the racket downwards (squat; down) and upwards (squat; up/rise to toes). | The algorithm sets playing time and minimum range of motion to squat movement based on players' body dimensions. The squat movement must be performed in velocity set in the algorithm. | Time  Scope of movement  Velocity (Slow → Moderate → Fast) | |
| Balance/ Coordination | 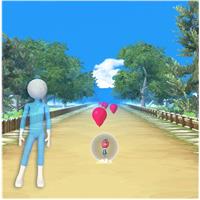 | BubbleRunner  The player moves the Avatar (figure inside the bubble) with weight transfer from side to side and tries to burst balloons by hitting them. | The algorithm sets playing time and minimum range of motion to side-to-side movement. | Time  Scope of movement | |
|  | 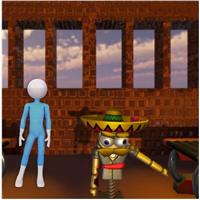 | HatTrick  The player moves the Avatar (figure with sombrero) by weight transfer from side to side and with hands tries to grasp the objects falling from the straps and throw them into a sombrero. | The algorithm sets playing time and minimum degree value to the side-to-side movement. The side-to-side movement must be performed in velocity set in the algorithm. | Time  Scope of movement  Velocity (Slow → Moderate → Fast) | |
|  | 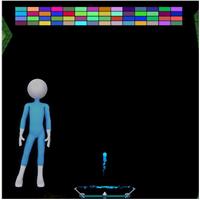 | BrickBreaker  The player moves the trampoline by weight transfer from side to side and bounces the ball in it so that the ball breaks the bricks at the top. The player can also capture falling fruits to the trampoline. | The algorithm sets playing time and minimum degree value to the side-to-side movement. | Time  Scope of movement | |
| Stretching | 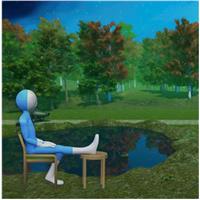 | Cannon  The player stretches the operated knee while doing target shooting with the cannon. Aiming and shooting of the cannon are done by hand movements. | The algorithm sets playing time. | Time | |
| Functional | 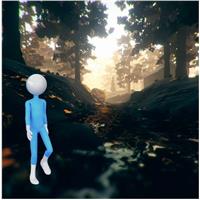 | Hiking  The player moves along the landscape path while walking (by lifting knees) in place. | The algorithm sets playing time and minimum percentage value to the walking movement. | Time  Scope of movement | |
|  | 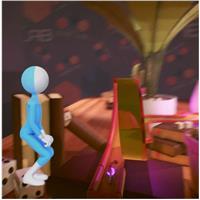 | ToyGolf  The player golfs by moving the Avatar (golfer) by weight transfer from side to side (targeting) and hitting golf swings with hand movements. The player also makes squats to move objects on the track (e.g., spin the windmill to give speed to the golf ball). | The algorithm sets playing time and range of motion to swing and squat movements based on players' body dimensions. | Time  Scope of movement | |

**3.**

**Standard home exercise protocols instructed to total knee replacement patients**

| **Turku University hospital** | |
| --- | --- |
|  | |
| Blood circulation, swelling (once in an hour minimum 20 reps) | |
|  | Supine or sitting: Bend and extend the ankles vigorously at a brisk pace. |
| Several weeks forward from the first post-operative day (3–5 times per day, 5–10 reps) | |
| Knee extension-flexion mobility | |
|  | Supine: Bend the operated knee as far as possible by sliding the foot along a bed. |
|  | Sitting: Bend the operated knee as far as possible, first by sliding the foot along the floor and later by assisting the movement with the other foot. |
|  | Sitting: While having the operated leg on another chair, allow the back of the knee to stretch. |
| Muscle strength | |
|  | Standing: Take support, bend the operated knee and move the heel towards the buttock. Hold for a moment and slowly lower down. Keep the thighs in line during the movement. |
|  | Supine: Extend the knee straight with the ankle flexed and lift the entire lower limb slightly off the bed, hold for a moment, and slowly lower down. |
| Walking | |
|  | Standing: Take a step forward with the operated lower limb extend the knee straight while transferring weight to the operated lower limb. |
| **Central Finland Central Hospital** | |
|  | |
| Six weeks forward from the first post-operative day (2 times per day, 3–10 reps, 1–3 sets) | |
| Knee extension-flexion mobility | |
|  | Sitting: Bend the operated knee as far as possible by sliding the foot along the floor. Hold for 15–30 seconds in the extreme position. |
|  | Sitting: While having the operated leg on another chair, allow the back of the knee to stretch for 20–30 seconds. |
| Muscle strength | |
|  | Sitting: While having a pillow under the operated knee, extend the knee as straight as possible without the back of the knee coming off the pillow, hold for a moment, and slowly lower down. Do the exercise also without the pillow just by tensing the thigh muscle. |
|  | Sitting: Extend the operated knee as straight as possible and slowly lower down. |
| Walking | |
|  | Standing: Stand with the feet side by side (take support from crutches). Take steps back and forward with the non-operated lower limb. Do the same with the operated lower limb. |
| From six weeks onwards | |
| Knee extension-flexion mobility (2–3 times per day, 3–5 reps, 20–30 sec/stretch, 1–3 sets) | |
|  | Standing: While the hands are on the wall, step back with the operated lower limb and stretch the operated knee straight. |
|  | Sitting: While the operated lower limb is extended, gently press the knee straight with the hands. Keep the back straight. |
|  | Standing: Lift the leg of the operated lower limb onto the chair and flex the knee by moving body weight forward. |
| Muscle strength (2–3 times per week, 10–15 reps, 2–3 sets) | |
|  | Standing: Step up to the first stair and step down. Do the exercise with both the lower limbs. Take support if needed. |
|  | Standing: Lean the back against the wall, feet off the wall at thigh length. Squat until thighs are at maximum parallel to the floor and get up. Take support if needed. |
|  | Sitting: Stand up and then slowly sit down. |
|  | Standing: Rise to the toes and slowly lower the heels down. |

**4.**

**Gym instructions were given to TKR patients at four-month post-operative research assessment visit** (8–12 reps, 3 sets)

| Exercise target | Gym equipment | Exercise instruction |
| --- | --- | --- |
|  |  |  |
| Lower body | Leg Press Machine | Place the feet on the footboard shoulder-width apart. When exhaling, extend your knees almost straight. Hold for a moment. Return to the starting position while inhaling. |
| Front of the thigh (quadriceps) | Leg Extension Machine | Extend the legs straight one at a time or at the same time. Slowly return to the starting position. |
| Back of the thigh (hamstring) | Leg Curl Machine | Bend the knees. Slowly return to the starting position. |
| Back of the shin (calf) | Calf Machine | Get up on the toes by extending the ankles and lifting the heels. Hold for a moment. Slowly return to the starting position. |
| Shoulders, back (latissimus dorsi) | Lat Pulldown Machine | Hold the bar by the shoulder-width grip. Pull the bar close to the chest or behind the neck. |
| Back (latissimus dorsi) | Seated Row Machine | Pull the arms towards the chest, keeping the elbows attached to the sides. Keep the abdominal muscles tight during the exercise. |
| Back (extensors) | Back Extension Machine | Extend the back (with caution if back problems exist). |
| Abdomen | Abdominal Crunch Machine | Bend the body forward with the abdominal muscles. Do not use the arms to perform the movement. |
