# Supplementary material for: Effectiveness of Gamification in Knee Replacement Rehabilitation: Protocol for a Randomized Controlled Trial With a Qualitative Approach
Source: JMIR Res Protoc. 2022 Nov 28;11(11):e38434. doi: 10.2196/38434 (PMC9745648; doi:10.2196/38434)
Supplement: Multimedia Appendix 2 [file resprot_v11i11e38434_app2.pdf]

VARSINAIS-SUOMEN
SAIRAANHOITOPIIRI

Polven tekonivel-leikkaus

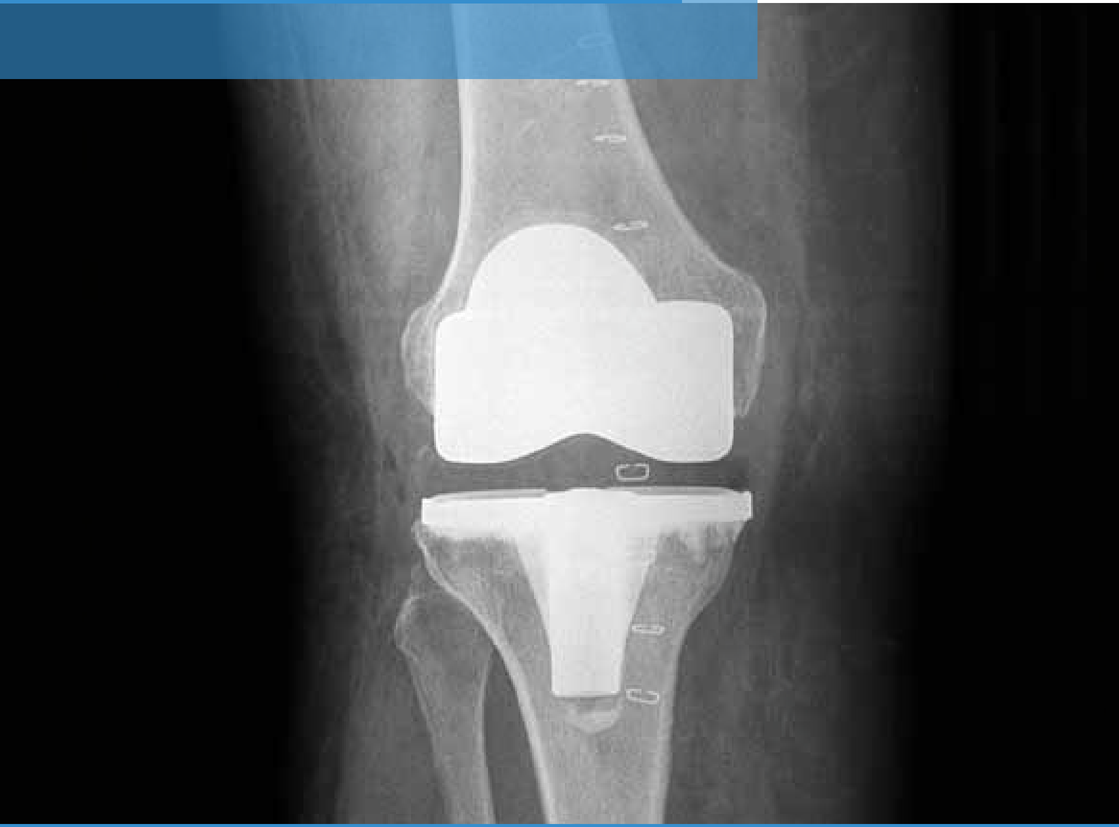

OHJEITA LEIKKAUKSEEN TULEVALLE POTILAALLE

Julkaisija: Varsinais-Suomen sairaanhoitopiiri Toimitus: Tyks Tules-toimialue
Ulkoasu ja taitto: Letterhead / Tuulevi Paakkanen Valokuvat: VSSHP:n arkisto
Paino: SpinPress 2015, Tallinna, Viro

Opas sinulle, joka olet tulossa polven tekonivelleikkaukseen

1. Polven nivelrikko . . . 3
2. Ennen leikkaushoitoon tuloa huomioitavat asiat . . . 4
   2.1. Tulehdusten hoito . . . 4
   2.2. Hampaiston hoito . . . 4
   2.3. Sairaudet ja lääkitykset . . . 5
   2.4. Ravitsemus . . . 5
   2.5. Lihasvoima . . . 5
   2.6. Apuvälineet . . . 6
   2.7. Tupakointi . . . 6
   2.8. Alkoholi . . . 6
   2.9. Muuta huomioitavaa . . . 6
3. Leikkaukseen valmistava käynti . . . 6
4. Leikkaus . . . 7
5. Leikkauksen jälkeen . . . 8
   5.1. Laskimotukosten ennaltaehkäisy . . . 8
   5.2. Leikkauksen jälkeinen asentohoito . . . 8
   5.3. Sauvakävelyohjeet . . . 8
   5.4. Harjoitusohjelma leikkauksen jälkeen . . . 7
   5.5. Jatkokuntoutus . . . 11
6. Sairaalavaiheen jälkeen . . . 12
   6.1. Leikkausalueen tarkkailu kotona . . . 12
   6.2.Tulehdusten ehkäisy ja hoito . . 12
   6.3. Liikunta . . . 13
   6.4. Seksuaalielämä ja raskaus . . 14
   6.5. Tekonivel ja metallinilmaisimet . . . 14
7. Tekonivelen irtoaminen . . . 14

Olet tulossa polven tekonivelleikkaukseen. Tämän oppaan tarkoituksena on auttaa sinua valmistautumaan leikkaukseen. Tästä oppaasta löydät tietoa leikkaukseen valmistautumisesta, leikkauksesta, sekä leikkauksen jälkeisestä hoidosta ja kuntoutuksesta.

1. Polven nivelrikko

Polven tekonivelleikkauksen yleisin syy on polven nivelrikko eli kuluma. Nivelrikko kehittyy tavallisesti ikääntymisen myötä ilman erityistä syytä. Perinnöllisillä tekijöillä on kuitenkin selvä merkitys nivelrikon kehittymisessä. Synnynnäiset sairaudet, kasvukauden sairaudet, polven alueen vammat ja tulehdukset sekä reumataudit voivat myös johtaa nivelrikkoon. Luuston haurastuminen eli osteoporoosi ei sen sijaan vaikuta nivelrikon syntymiseen.

Polven nivelrikko aiheuttaa polven ja säären alueen kipua, joka voi säteillä kohti nilkkaa. Aluksi kipu tuntuu lähinnä liikkeelle lähtiessä ja vähitellen kävely alkaa vaikeutua. Nivelrikon edetessä monille potilaille tulee vähitellen lepo- ja yösärkyä. Nivelrikon takia kävely vaikeutuu ja polven liikkeet rajoittuvat. Vähitellen voi kehittyä virheasento tai ojennusvaje. Hoitamattomana nivelrikko voi johtaa nivelen jäykistymiseen.

Alkuvaiheessa kipuja voidaan lievittää vähentämällä polven kuormitusta esimerkiksi kävelykepin tai kyynärsauvojen avulla tai rasitusta vähentämällä. Särkylääkkeillä ja fysioterapialla voidaan lievittää oireita. Laihduttaminen helpottaa useimpien potilaiden kohdalla alkuvaiheessa oireita selvästi. Nivelrikon luonteeseen kuuluu oireiden vaihtelu ja alkuvaiheessa oireet voivat hävitäkin välillä. Ajan myötä kivut pyrkivät lisääntymään.

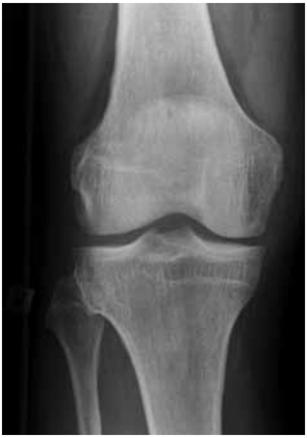

Yläkuvassa hyvin säilynyt polvi, alla kulunut, nivelrikon syömä polvinivel

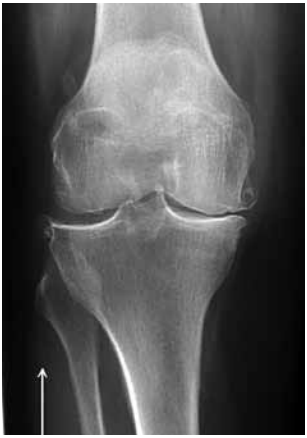

Polven tekonivelleikkaus on ajankohtainen, kun polvessa on merkittävää kipua ja kävely on selvästi rajoittunut eikä muilla hoidoilla oireisto enää korjaannu. Onnistunut tekonivelleikkaus tuo hyvän avun vuosiksi. Särky jää yleensä pois ja nivelrikon aiheuttamat kävelyvaikeudet korjaantuvat. Myös nivelen liikkuvuus paranee. Lihaskunnon palautuminen vaikuttaa oireiden korjaantumiseen ja usein toipumista tapahtuu pitkään, yli vuoden ajan leikkauksen jälkeen.

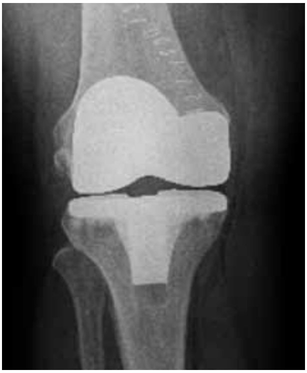

vaurioituneet nivelpinnat on poistettu ja korvattu tekonivelellä eli endoproteesilla.

2. Ennen leikkaushoitoon tuloa huomioitavat asiat

2.1. Tulehdusten hoito

Kaikki tulehdukset kuten esimerkiksi virtsatietulehdus, poskiontelotulehdus, tulehtunut ihottuma ja ihorikot voivat olla esteenä leikkauksen suorittamiselle. Siksi sinun on hoidettava ne ennen leikkausta. Tarvittaessa on hyvä ottaa yhteys myös jalkahoitajaan. Käynnit jalkahoitajalla suositellaan tehtäväksi viimeistään pari viikkoa ennen leikkausta.

2.2. Hampaiston hoito

Koko suun tarkastus ja hoito pitäisi tehdä riittävän ajoissa ennen tekonivelleikkausta. Hampaiston tulehduspesäkkeiden tiedetään aiheuttavan ajoittaista bakteerikylvöä verenkiertoon. Tulehduspesäkkeitä voi olla suun limakalvolla, ikenissä, hampaistossa sekä leukaluussa, jopa hampaattomassa leukaluussa.

Tulehduspesäkkeet voivat olla piileviä siten, että ne voidaan havaita ainoastaan röntgenkuvauksella. Ennen tekonivelleikkausta tulee hampaistosta ottaa röntgenkuva (ortopantomogrammi). Myös hampaattomat leuat tulee röntgenkuvata piilevien, leukaluun sisällä olevien tulehduspesäkkeiden tai jäännösjuurten havaitsemiseksi.

Tulehduspesäkkeiden hoito tulee suunnitella tehtäväksi hyvissä ajoin ennen tekonivelleikkausta. Esimerkiksi hampaan poistokuopan paranemiselle olisi varattava vähintään neljä viikkoa.

Hampaiston kiinnityskudoksen terveyteen tulee kiinnittää erityistä huomiota. Ientulehdus voi olla huomaamatta levinnyt laajalle hampaistossa ja sen kiinnityskudoksissa. Ientulehduksen oikeaoppinen hoito vaatii aikaa ja omaa aktiivisuutta. Tämän vuoksi sinun tulee hakeutua hammaslääkärin vastaanotolle hyvissä ajoin ennen tekonivelleikkausta. Tutkimukset ja hoito on syytä aloittaa heti kun leikkauspäätös on tehty. Hammaslääkärin lausunto suun terveydentilasta on voimassa 6 kuukautta.

2.3. Sairaudet ja lääkitykset

Suurimman riskin leikkauksille aiheuttavat sepelvaltimosairaudet ja aivoverenkiertohäiriöt.

Verenpainetauti ja sokeritauti tulisi olla hyvässä tasapainossa leikkaukseen tultaessa. Käy tarvittaessa kontrollissa ja lääkitysten tarkistuksessa omalla terveysasemalla n. 1–2 kuukautta ennen leikkausta. Mikäli käytät säännöllisesti Marevania® tai muita verenohennuslääkkeitä tai jos käytössäsi on biologisia lääkkeitä, muista kertoa siitä hoitohenkilökunnalle.

2.4. Ravitsemus

Monipuolinen ravinto ja nesteiden nauttiminen leikkausta edeltävinä viikkoina auttavat sinua toipumaan paremmin leikkauksesta. Se vahvistaa elimistön puolustusjärjestelmää ja tulehdusten riski vähenee.

Ylipaino vaikeuttaa leikkauksen teknistä suorittamista, pitkittää toipumisaikaa ja lisää tekonivelen irtoamisriskiä. Lisäksi ylipaino lisää komplikaatioriskiä leikkauksen aikana ja voi myös estää leikkaukseen pääsyn. Painoa voit pudottaa muuttamalla ruokailutottumuksiasi. Painonpudotus on syytä aloittaa hyvissä ajoin ennen leikkausta. Tarvittaessa kotipaikkakunnan terveyskeskus antaa laihdutus- ja ruokavalio-ohjeita.

2.5. Lihasvoima

Polviniveltä liikuttavien lihasten hyvä kunto sekä hyvä yleiskunto edesauttavat leikkauksesta toipumista. Tässä ohjeessa olevia harjoitusliikkeitä on hyvä tehdä kotona nivelten liittuvuuden ja lihasvoiman ylläpitämiseksi. Pyöräily, uinti, kävely sekä talvisin murtomaahiihto ovat polven nivelrikkopotilaalle suositeltavia liikuntamuotoja.

2.6. Apuvälineet

Leikkauksen jälkeen tulet alussa liikkumaan kyynärsauvojen avulla. Sinun tulee hakea ne ennen leikkaukseen tuloa omasta terveyskeskuksesta/apuvälineyksiköstä.

2.7. Tupakointi

Tupakointi supistaa verisuonia ja hidastaa luutumista. Suosittelemme tupakoinnin lopettamista tai ainakin vähentämistä hyvissä ajoin ennen leikkausta, viimeistään kuukautta ennen leikkausta, jotta siitä olisi hyötyä. Tarvittaessa voit ottaa yhteyttä tupakointivieroitusryhmään tai käyttää apuna nikotiinilaastareita tai -purukumia.

2.8. Alkoholi

Alkoholin runsas käyttö tulee lopettaa hyvissä ajoin ennen leikkausta. Vieroitusoireet vaikeuttavat leikkauksesta toipumista ja kuntoutumista. Runsas alkoholinkäyttö voi myös estää tekonivelleikkaukseen pääsyn.

2.9. Muuta huomioitavaa

Jos koet, että leikkauksen jälkeen kotona selviytymisessä saattaa olla ongelmia, voit tiedustella oman kuntasi kotipalvelusta mahdollista kotiapua. Myös sairaalan sosiaalityöntekijä auttaa tarvittaessa avun järjestämisessä.

3. Leikkaukseen valmistava käynti

Noin kaksi viikkoa ennen leikkausta sinut kutsutaan leikkaukseen valmistavalle käynnille. Tämän käynnin yhteydessä tai etukäteen terveyskeskuksessa sinusta otetaan tarvittavat laboratoriokokeet ja röntgenkuvat. Käynnillä tapaat endoproteesihoitajan sekä tarvittaessa leikkaavan lääkärin, fysioterapeutin ja anestesialääkärin. Heiltä saat tietoa leikkaukseen liittyvistä asioista.

Toimenpiteen jälkeen tarvitset kyynärsauvat. Voit hakea ne lainaksi oman kotikuntasi terveyskeskuksen apuvälinelainaamosta. Ota ne mukaan tullessasi leikkaukseen.

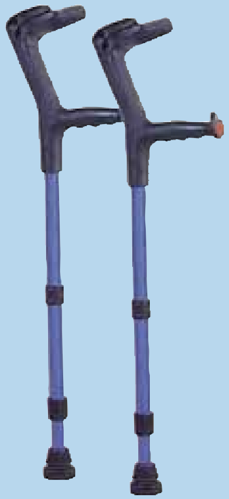

Turun sosiaali- ja terveystoimi, Apuvälineyksikkö

Luolavuorentie 2,
Rakennus 1 G, Turku

avoinna:
ma, ti, to, pe klo 9–15
ja ke klo 9–17
(heinäkuu ke klo 9–15)

p. 266 2264

Muiden kuntien apuvälinelainaamoiden osoitteet ja aukioloajat saat selville omasta terveyskeskuksesta.

4. Leikkaus

Polven tekoniveleen kuuluu reisiosa, sääriosa sekä tarvittaessa polvilumpio-osa. Proteesin reisiosa on aina metallia. Proteesin sääriosa on polyeteenimuovia ja metallia. Polven tekonivel kiinnitetään nykyisin luuhun aina luusementillä, joka kovettuu noin 15 minuutissa leikkauksen aikana. Proteesi kestää kuormittamisen heti, eli saat varata leikatulle raajalle alusta lähtien

Tekonivelleikkaus tehdään useimmiten selkäpuudutuksessa ja leikkaus kestää tavallisesti 1–2 tuntia.

Leikkauksen jälkeiseen kivun hoitoon on erilaisia menetelmiä, joista valitaan sinulle sopivin. Kivun hoidoksi leikkaava lääkäri voi laittaa leikkauksen aikana polviniveleen ja sitä ympäröiviin kudoksiin lääkeseosta, joka sisältää tavallisimmin kipu- ja puudutusainetta. Lisäksi jalkaan laitetaan tukisidos, joka poistetaan seuraavana aamuna. Toisinaan kipua hoidetaan muutaman päivän ajan selkään laitettavan kipukatetrin avulla. Lisäksi aloitetaan tablettilääkitys, joka jatkuu myös kotona säännöllisesti 1–2 kuukauden ajan helpottamaan harjoitusohjelman läpivientiä. On tärkeää, että kerrot kivustasi henkilökunnalle.

Leikkauksen jälkeen käytät kävelyssä apuna kyynärsauvoja tavallisesti 1–2 kuukauden ajan.

Polven proteesileikkauksen jälkeen sairaalahoito kestää yleensä 3–4 päivää, polven osaproteesin jälkeen yleensä 2–3 päivää. Kotiutumisen edellytyksenä on, että liikkuminen on turvallista ja omatoimista, leikkaushaavan paraneminen on alkanut ja kivut ovat hallinnassa kipulääkkeillä. Leikkauksen jälkeinen aika vaatii sinulta aktiivista osallistumista harjoitteluun mahdollisimman hyvän liikelaajuuden saavuttamiseksi.

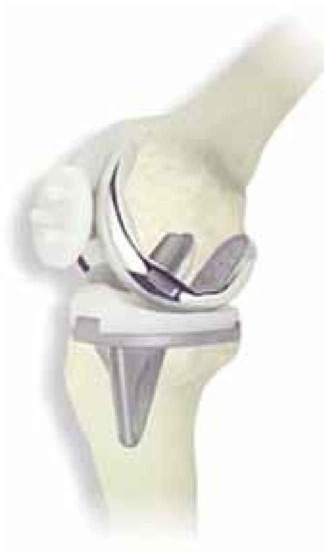

polviproteesi

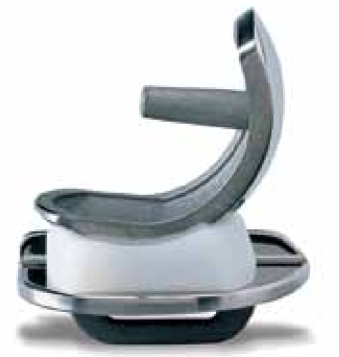

osaproteesi

5. Leikkauksen jälkeen

5.1. Laskimotukosten ennaltaehkäisy

Leikkauksen jälkeen liikunta on erittäin tärkeää laskimotukosten ehkäisemiseksi. Osastolla aloitetaan laskimotukoksia ja keuhkoveritulppaa ehkäisevä lääkitys, jota jatkat vielä kotonakin.

5.2. Leikkauksen jälkeinen asentohoito

Kylmäpakkaus tms. ja kohoasento vähentävät leikatun jalan turvotusta ja kipua. Pidä kotonakin tarvittaessa jalkaa kohoasennossa useita kertoja päivässä. Muista pitää polvitaive tyhjän päällä. Kylmäpakkausta on hyvä pitää useita kertoja päivässä noin 15 minuuttia kerrallaan.

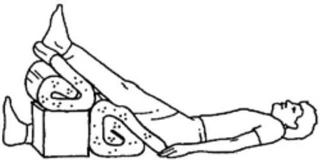

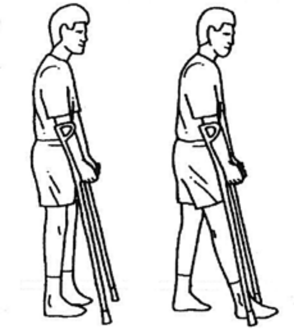

5.3. Sauvakävelyohjeet

Ylösnousu- ja kävelyharjoitukset aloitetaan fysioterapeutin tai/ja hoitajan avustamana jo leikkauspäivän iltana.

Aseta sauvat eteen.
Astu leikatulla jalalla sauvojen väliin.
Astu terveellä leikatun jalan ohi.
Ota yhtä pitkät askeleet molemmilla jaloilla.

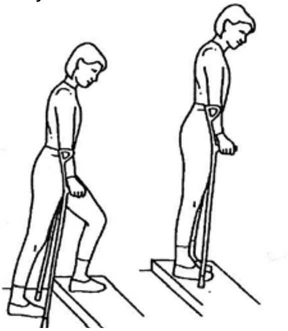

Kävelyn varmistuttua harjoitellaan myös porraskävely:

Porraskävely ylös
Astu terveellä jalalla ylemmälle portaalle.
Nosta sitten leikattu jalka ja sauvat samalle portaalle.
Noin kuukauden kuluttua voit nousta portaat normaalisti vuorotahtiin.

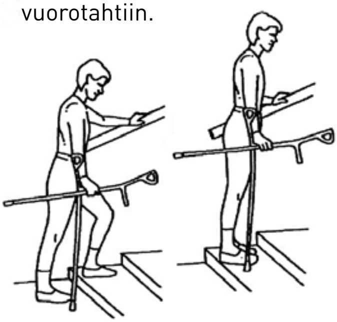

Portaat ylös kaiteesta kiinni pitäen.
Astu terveellä jalalla porras ylös, nosta sitten leikattu jalka ja sauva samalle portaalle.

Porraskävely alas
Laske sauvat ja leikattu jalka alemmalle portaalle, vie terve jalka samalle portaalle.

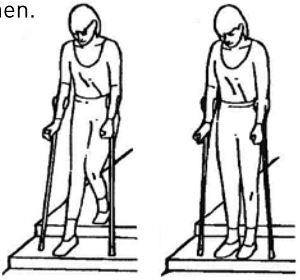

5.4. Harjoitusohjelma leikkauksen jälkeen

Harjoitusohjelman tarkoituksena on vähentää turvotusta ja vilkastuttaa verenkiertoa sekä parantaa polvinivelen liikkuvuutta ja jalan lihasten toimintaa. Harjoittelu aloitetaan ensimmäisenä leikkauksen jälkeisenä päivänä.

Sinun tulisi tehdä harjoituksia useita kertoja päivässä ensimmäiseen jälkitarkastukseen asti. Kertamääriä voit lisätä tuntemusten mukaan. Harjoittelu on helpompaa, jos olet pitänyt kylmäpakkausta sitä ennen polven päällä. Suorita liikkeet rauhallisesti. Rentoudu liikkeiden välillä.

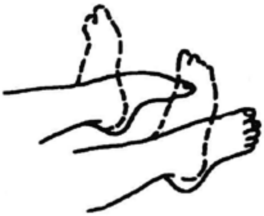

1. Selinmakuulla.
Koukista ja suorista nilkkoja reippaasti, 20 kertaa/tunti.

Piirroskuvat: Physio Tools

---

2. Selinmakuulla.
Vedä nilkat koukkuun ja paina samanaikaisesti vain polvitaipeet tiukasti alustaa vasten.
Pidä jännitys 5 sekuntia – rentoudu.
Yritä saada nilkat nousemaan kevyesti irti alustalta.
Toista ____ kertaa.

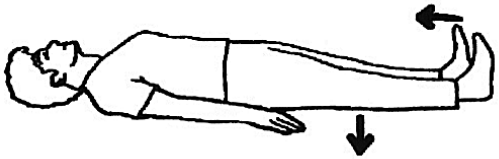

---

3. Selinmakuulla.
Koukista leikattu jalka mahdollisimman pitkälle alustaa pitkin.

Toista 5–____kertaa.

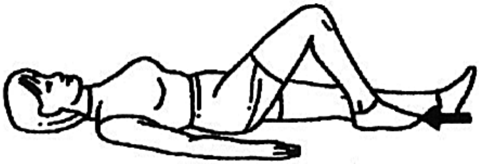

---

4. Selinmakuulla rulla polvien alla.
Ojenna leikattu jalka mahdollisimman suoraksi nilkka koukussa, pidä hetki ja laske rauhallisesti alas.
Ojennuksen aikana polvi ei saa nousta rullan päältä pois.

Toista 5–____kertaa.
Tee myös toisella jalalla.

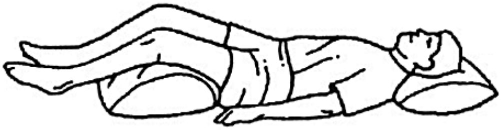

Piirroskuvat: Physio Tools

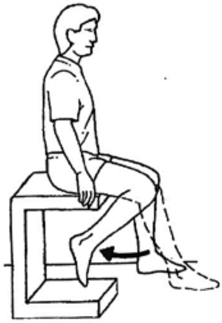

5. Istuen
Koukista polvea mahdollisimman pitkälle.
Alkuun voit tehdä liikkeen jalkapohja alustalla,
myöhemmin istuen korkealla jalat roikkuen,
avustaen koukistusta toisella jalalla.

Toista 5–10 kertaa.

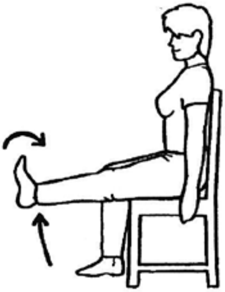

6. Istuen
Ojenna polvi suoraksi nilkka koukussa.
Pidä reisilihasjännitys hetken ja laske hitaasti alas.

Toista 5–___ kertaa.

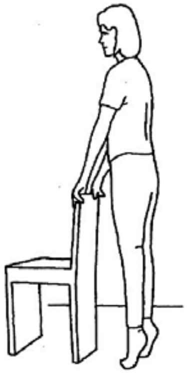

7. Seisten
Ota kiinni jostain tukevasta,
esim. kaiteesta tai tuolin selkämyksestä.
Nouse varpaille.

Toista 10–___ kertaa.

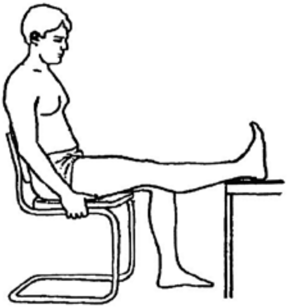

8. Istu tuolilla harjoitettava jalka
tuettuna kuvan mukaisesti.
Anna polvitaipeen venyä tässä asennossa.

Pidä asento ___ min, __ kertaa päivässä.

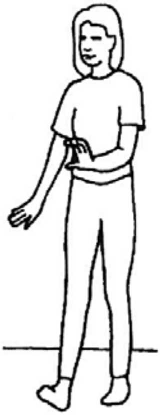

9. Seisten
Ota askel leikatulla jalalla eteen, ojenna polvi suoraksi siirtäen samalla painoa leikatulle jalalle.

Toista 5–10 kertaa.

---

10. Selinmakuulla, toinen jalka hieman koukussa.
Ojenna polvi suoraksi nilkka koukussa ja nosta koko jalka hieman ylös alustalta.
Pidä jännitys hetken ja laske jalka hitaasti alustalle.
Rentoudu.

Toista 5–__kertaa.

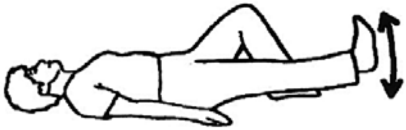

---

11. Seisten selkä suorana.
Ota tukea ja vie jalka hieman taaksepäin.
Vedä kantapäätä kohti takamusta. Pidä jännitys hetken.
Pidä liikkeen aikana reidet samassa linjassa.

Toista ___ kertaa

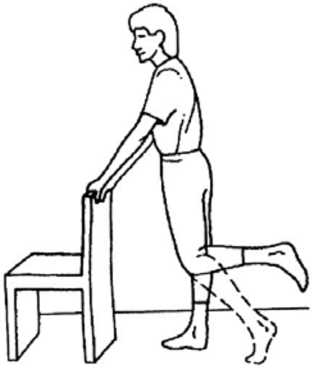

Piirroskuvat: Physio Tools

5.5. Jatkokuntoutus

Kuntoutuminen edellyttää omaa aktiivista päivittäistä harjoittelua. Pääsääntöisesti fysioterapiakontrolli toteutetaan oman kunnan terveyskeskuksen fysioterapiassa 4–6 viikon kuluttua toimenpiteestä. Saat fysioterapiaan kutsun postitse. Vesiliikunnan saa aloittaa aikaisintaan 6 viikon kuluttua.

6. Sairaalavaiheen jälkeen

6.1. Leikkausalueen tarkkailu kotona

Noudata kotiutumisen yhteydessä annettuja haavanhoito-ohjeita.

Haavalla olevat hakaset poistetaan terveyskeskuksessa tai kotisairaanhoitajan toimesta 2 viikon kuluttua leikkauksesta: ____________________________. Haavalla voi olla myös sulavat ompeleet, jolloin niitä ei tarvitse poistaa.

Älä kastele haavaa mikäli se erittää vielä verta. Haavaa ei saa suihkuttaa paineella, saippuoida tai hangata. Jos haavalta vuotaa verta tai muuta eritettä, peitä se puhtaalla haavasidoksella. Vaihda likaantunut tai kastunut haavasidos. Huolehdi hyvästä käsihygieniasta haavanhoidossa. Älä koske haavaan turhaan.

Kuivalta haavalta sidokset saa poistaa ja suihkuun mennä kun toimenpiteestä on kulunut 5 vuorokautta. Saunaan saat mennä vuorokauden kuluttua ompeleiden/hakasten poistosta. Kylpyyn tai uimaan saat mennä kuuden viikon kuluttua leikkauksesta.

Jos leikkausalueella tuntuu äkillistä, pahenevaa kipua, leikkaushaavassa esiintyy punoitusta, turvotusta tai vuotoa tai kotona on jatkuvaa kuumeilua (yli 38°), ota yhteyttä sinua hoitaneeseen osastoon. Leikatun jalan turvotus on tavallista, mutta jos siihen liittyy pohjekipua eikä turvotus häviä levon aikana, ota yhteyttä osastoon

6.2. Tulehdusten ehkäisy ja hoito

Tekonivel on vierasesine kehossa ja siksi alttiimpi tulehduksille kuin muu kudos. Siihen voi myöhemmin tulla tulehdus muualta elimistöstä. Siksi infektioiden ennaltaehkäisy ja hoito on tekonivelleikkauksen jälkeen tärkeää. Kaikki tulehdussairaudet, esimerkiksi hammasjuuritulehdukset ja keuhkoihin ja virtsateihin liittyvät tulehdukset tulee hoitaa huolellisesti. Normaalit virusperäiset hengitystietulehdukset, esimerkiksi tavalliset flunssat, eivät ole tulehdusriski tekonivelen kannalta. Erityisesti kroonista niveltulehdusta sairastavilla potilailla (esim. nivelreuma, nivelpsoriasis), jotka joutuvat sairauden vuoksi pitkäaikaisesti käyttämään kortisoni- tai solunsalpaajahoitoa, voi proteesin tulehtumisriski olla suurentunut.

Lääkärissä ja hammaslääkärissä käynnin yhteydessä sinun on aina ilmoitettava tekonivelestä.

Hampaan poiston ja muiden verenvuotoa aiheuttavien toimenpiteiden yhteydessä infektiokykyiset suun bakteerit pääsevät verenkiertoon. Tämä ohimenevä bakteremia kestää n. 20 minuuttia. Suun limakalvoa rikkovien toimenpiteiden yhteydessä suusta peräisin olevat bakteerit voivat kiinnittyä tekoniveleen, jolloin se saatetaan joutua jopa poistamaan. Antibioottisuoja on otettava ennen toimenpidettä. Myös nenän, nielun, hengitysteiden, vatsasuolikanavan, virtsatie- ja sukuelinten alueiden toimenpiteissä suositellaan antibioottiprofylaksiaa.

Tavanomaisissa, ei-invasiivisissa hammashoitotoimenpiteissä (esimerkiksi hampaan paikkaus) antibioottiprofylaksiaa ei tarvita. Ensimmäisen 6 kk:n aikana tekonivelen asentamisen jälkeen on pyrittävä välttämään bakteerien veriteitse leviämistä aiheuttavia toimenpiteitä.

Suun kautta annettava profylaksiasuositus aikuisilla:
Amoksisilliini 2g kerta-annos tunti ennen toimenpidettä
penisilliiniallergisilla Klindamysiini 600mg kerta-annos

6.3. Liikunta

Tekonivel kestää hyvin normaalia päivittäistä liikkumista. Alkuvaiheessa kuntoiluksi suositellaan kävelyä sisällä ja ulkona. Uinti, vesivoimistelu, pyöräily, kävely ja kuntosaliharjoittelu ovat suositeltavia sekä lihasten vahvistamiseksi että nivelen liikkuvuuden lisäämiseksi. Hyötyliikunta on erinomainen liikuntamuoto tekonivelpotilaalle. Hyötyliikuntaa ovat esim. puutarhanhoito, nurmikonleikkuu ja lumityöt. Hölkkä, juokseminen ja pallopelit ovat lajeja, joissa paino tulee tärähtäen leikatun jalan päälle. Toistuva iskumainen kuormitus voi johtaa tekonivelen ennenaikaiseen kulumiseen ja irtoamiseen. Liikuntaharrastusten aloittamisesta sinun on hyvä keskustella hoitavan lääkärin ja fysioterapeutin kanssa.

Liukkaalla talvikelillä liikkuessasi sopivia apuvälineitä ovat kenkiin kiinnitettävät liukuestenastat ja kyynärsauvoihin kiinnitettävät jääpiikit.

6.4. Seksuaalielämä ja raskaus

Seksuaalisuus on osa elämää, ihmisyyttä, sukupuolisuutta, minäkuvaa ja kehonkuvaa. Sairaudet, vaivat ja niiden hoidot voivat vaikuttaa seksuaaliterveyteesi. Ota rohkeasti puheeksi, jos haluat kysyä ja keskustella seksuaaliseen hyvinvointiisi liittyvistä asioista lääkärin tai hoitajan kanssa. Raskaus ja synnyttäminen on mahdollista tekonivelleikkauksen jälkeen. Seksuaalielämälle ei ole rajoituksia, joskin paranemassa oleva leikkaushaava on huomioitava.

6.5. Tekonivel ja metallinilmaisimet

Metallinilmaisimet voivat reagoida tekoniveleen. Nykyisin erillistä todistusta ei lentokentällä tarvita. Matkailua tekonivelet eivät estä.

7. Tekonivelen irtoaminen

Nykyiset tekonivelet kestävät varsin hyvin. Tekonivelen irtoaminen on mahdollista, jolloin voidaan joutua tekemään uusintaleikkaus. Irtoamisriski kasvaa vähitellen. Irtoamisriskin takia leikatun polven seuranta on tärkeää lääkärin kanssa sovittavan aikataulun mukaan. Pääsääntöisesti kontrollit tapahtuvat omassa terveyskeskuksessa 3–5 vuoden välein. Kontrollien toteutumisesta on huolehdittava itse.

MUISTILISTA:

**Hampaat**	☐	hampaiston röntgenkuvaus (ortopantomogrammi)
	☐	hammaslääkärin tutkimus ja hoito
	☐	hammaslääkärin lausunto
**Iho**	☐	iho terve ja ehjä, ei ihottumia
	☐	ihopoimut ja varvasvälit ehjät
	☐	ei säärihaavoja sekä kynnet hoidettu
**Infektiot**	☐	kaikki tulehdukset hoidettu ennen tekonivelleikkausta (esim. virtsa-, poskiontelo- ja hammastulehdukset)
	☐	tarvittaessa yhteys oman terveyskeskuksen lääkäriin
**Sairaudet**	☐	perussairaudet tasapainossa ennen leikkausta esim. diabetes, verenpainetauti ym.
	☐	tarvittaessa yhteys oman terveyskeskuksen lääkäriin
**Lääkitys**	☐	lääkityksen tarkistus omalla terveysasemalla
**Lihasvoima**	☐	hyvä yleiskunto sekä lihasten hyvä kunto (esim kävely, pyöräily, uinti)
**Ravitsemus**	☐	monipuolinen ravinto
	☐	tarvittaessa painon pudotus
**Tupakointi**	☐	tupakoinnin lopettaminen / vähentäminen.

Ota tämä vihkonen mukaan sairaalaan

Potilaan nimi . . . .

Leikkaukseen valmistava käynti. . . . .

Leikkaava lääkäri. . . . .

Varausohjeet . . . .

Muut huomioitavat asiat . . . .

Kontrollikäynti . . . .

. . . .

. . . .

. . . .

. . . .

VARSINAIS-SUOMEN
SAIRAANHOITOPIIRI

Tyks Tules

TARVITTAESSA OTA YHTEYTTÄ:

- **Tyks Kirurginen sairaala,** tekonivelkirurgia
  arkisin klo 12-15 poliklinikka . . . . p. 02 313 6225
  muina aikoina vuodeosasto . . . . p. 02 313 6134
- **Tyks Kirurginen sairaala,** reumaortopedia
  ma klo 8-8.30, ti, ke ja pe klo 8-10 . . . . p. 02 313 4316
  muina aikoina vuodeosasto . . . . p. 02 313 6180
- **Tyks Kirurginen sairaala,** artron vastuualue . . . . p. 02 313 6166
- **Tyks Salon sairaala**
  kirurgian yksikkö . . . . p. 02 314 4475
- **Tyks Loimaan sairaala**
  kirurgian poliklinikka . . . . p. 02 314 3954
  osasto 2 . . . . p. 02 314 3226
- **Tyks Vakka-Suomen sairaala**
  kirurgian osasto . . . . p. 02 314 1100
- **Turunmaan sairaala**
  kirurginen vuodeosasto . . . . p. 02 314 6280
  sairaanhoitaja, ma ja to klo 8.30–9.15 . . . . p. 02 314 6166

313302 PT 74s
