# Supplementary material for: Effectiveness of Gamification in Knee Replacement Rehabilitation: Protocol for a Randomized Controlled Trial With a Qualitative Approach
Source: JMIR Res Protoc. 2022 Nov 28;11(11):e38434. doi: 10.2196/38434 (PMC9745648; doi:10.2196/38434)
Supplement: Multimedia Appendix 3 [file resprot_v11i11e38434_app3.pdf]

Guidebook for knee replacement patients
Improving your quality of life

Guidebook for knee replacement patients

This guidebook is meant for patients undergoing examinations and preparing for a joint replacement surgery. This booklet will provide you with useful and important information on how to get ready for your surgery, the operation itself, and the recovery process. This information is vital for your health, as you need to take care of several things before the surgery.

Before you can be placed in que for surgery, we need to check your overall health. Your health centre will make sure your health is good enough so that your body can handle the operation and recovery process. Otherwise your surgery needs to be cancelled. An orthopaedic specialist at Central Finland Central Hospital will make the final decision with you.

Make sure you study this guidebook thoroughly.

Keep this guidebook and take it with you to all your appointments.

For more information on the surgery and support groups (in Finnish):

www.ksshp.fi/tekonivelleikkaus and www.nivel.fi

You can also download this guidebook on your mobile device.

Contents

3 Good overall health is a precondition

7 Visiting the surgery outpatient clinic

8 Preparing for your surgery

9 On the day of your surgery

10 After the surgery

17 At home after the surgery

19 Living with joint replacement

20 Checklist before surgery

21 Notes and your own questions

22 Important information on your surgery

23 Contact us

Good overall health is a precondition

Joint replacement surgery requires a lot of preparations. After your overall health has been checked and you are given a surgical clearance, your doctor will refer you to the surgical outpatient clinic in Hospital Nova. For a surgical clearance, the following things need to be taken care of.

1. Make sure your long-time diseases are under control

Long-time diseases should be under control, as that makes the surgery and recovery process easier for you. Your health centre will refer you to a nurse and a physiotherapist who will then check your current health, and if needed, schedule further examinations.

Examples on the importance of controlled long-time diseases:

Asthma and COPD. Controlled health of your lungs enhances the safety of the operation by minimizing risks and making the recovery process smoother.

- If needed, your nurse will refer you further examinations such as a pulmonary function test.

Diabetes. When your diabetes is under control, the wounds will heal quicker and the chance of infection is lower.

- Make sure to control your blood sugar. If the levels differ from your target range, contact the health centre in charge of your diabetic care.

Prostate enlargement

- Before surgery, men with prostate enlargement need to take care of any possible difficulties with urinating. Discuss the matter with your doctor.

Skin conditions and ulcers. Acute skin infections and infected rashes prevent the surgery, as bacteria on your skin may spread to the joint replacement via circulation and cause infections.

- Make sure your skin is as intact as possible before the operation. If your skin condition gets worse near the surgery, contact your health centre.

Rheumatoid arthritis. Your doctor will advise you to stop taking biologic drugs before the operation. This decreases the risk of infections.

- Make sure your arthritis is under control. If you have any problems with your condition, contact the provider of your rheumatic care.

Hypertension and coronary artery disease. Controlled situation enhances the safety of your operation as well as the recovery process.

2. Check your skin condition

Scratches, scabs, pimples, infected rashes, paronychia (inflammation of the skin around the nail), leg ulcers as well as intertrigo or ulcers between your toes will prevent your surgery.

To take care of your feet at home, wash and dry them thoroughly, and wear well-fitting cotton socks that are non-binding. Contact a nurse or a foot therapist if you notice any skin or nail deformities you cannot take care of yourself.

Before surgery:

- Check and take care of all possible skin infections such as intertrigo or ulcers between your toes, under your breasts, and in the inguinal region.
- Avoid scratching and rubbing your skin
- Avoid shaving during the last 7 days before your surgery.

Check your skin condition:

- Armpits
- Area of the surgery
- Area of the surgery
- Inguinal region
- Under your breasts
- Between your toes

3. Dental care

Make an appointment with a dentist. All problems with your mouth and teeth need to be treated before the surgery. Infections in your gums or anywhere in your mouth may be hidden or inactive (even if you have lost your teeth), and they can't be detected without a dentist's check-up and possibly X-ray.

4. Pay attention to nutrition and weight

Pay attention to nutrition by eating a well-balanced and healthy diet. Make sure you are getting enough vitamin D, calcium and protein. Good nutritional status will speed up your recovery process and improve your immune system. Overweight and malnutrition increase risks, slow down the recovery process, and shorten the life span of your joint replacement.

If you need advice or help with your diet or weight loss, consult a nutritionist or a nurse at your health centre. For more information on exercise groups, please contact a physiotherapist.

Make sure you stop taking any Omega-3 supplements and other natural health supplements at least two months before your surgery, as they increase the chance of bleeding during surgery.

5. Exercise regularly

Good overall health and strong muscles ease the symptoms of osteoarthritis, improve joint mobility and circulation, and speed up the recovery process after surgery. Make sure to exercise regularly and keep your exercise routine versatile.

Increase the amount of exercise little by little and use pain medication if needed. If the joint swells or feels substantially sore, try changing your exercise routine until the swelling or pain settles. If you need any help or support on exercise, please contact a physiotherapist at your health centre.

6. Take care of pain management

Make sure your pre-surgery pain management is taken care of. Cold therapy, exercise, rest, functional aids and a tailored pain medication help you get through your daily routines. A well-rounded pain management plan enables an active lifestyle. Both before and after your surgery, your doctor will ask you to describe your current pain level on a scale of 0–10.

7. Quit smoking

Quit using all tobacco products, including e-cigarettes, at least two months before your surgery.

Smoking will hinder your recovery and increase the risks related to anaesthesia and surgery:

- higher risk of blood clots
- surgery-related breathing problems are 5 times more likely
- wounds will heal more slowly and be more likely to caught infections
- immunity against all infections is weakened
- ossification is hindered

When you quit smoking:

- Decide on a day.
- If you wish to discuss replacement therapy or medication, contact your health centre.
- Seek help and support from your health centre, family members, friends, colleagues, withdrawal support groups, a psychologist, a pharmacy, or call a support line.
- (Stumppi support line, tel. 0800 148 484, Mon-Tue at 10:00-18:00, Thu at 13:00–16:00).
- Prepare for withdrawal symptoms. They will occur within 2–12 hours after your last cigarette. Withdrawal symptoms peak during the first weeks, and they can last from 3–4 weeks to several months.

8. Quit all substances

If you are under influence of alcohol or drugs, your surgery will be cancelled. It is strictly forbidden to use any substances during the last 24 hours before you come to the hospital.

You need to quit heavy or regular drinking and using any substances at least two months before your surgery. If you find quitting hard or you want support, please contact the substance abuse nurse in your health centre.

It is important that you quit all substances:

- the combined effect of alcohol, drugs and the medication used in the surgery may be fatal
- substances increase the risk of accidents
- withdrawal symptoms hinder the recovery process and rehabilitation

Alcohol as well as other substances make you more prone to accidents also during your recovery process.

9. Pay attention to mental well-being and sleeping

Mental well-being is a source of strength that will help you prepare for your surgery and recover after it. Pains, changes in your day-to-day life, and the surgery itself may leave you feeling nervous, scared or anxious. It's a good idea to discuss these feelings with your friends and family. You can also contact your health centre with these issues.

Take enough time for resting and sleeping. Getting enough good-quality sleep will help you stay active and able to go about your day-to-day life, as well as recover after your surgery.

10. Make plans with your family and friends

Tell your family and friends about your upcoming surgery, as their support is very important during all stages of the process. Discuss and plan what kind of support you may need at home while you recover from the surgery. Think about the support and help you might need with your day-to-day activities at home. It's good to plan ahead: who will take care of grocery shopping, who will cook, who will tidy the house?

Sometimes the help from family and friends is not available, or they cannot provide enough help. In this case, seek information on the support services available in your home city or municipality. A nurse at your health centre will be happy to provide you with more information on the available services. If you need help with financial issues, please contact a social worker.

11. Make sure your home is accessible

Plan and make necessary preparations to make your home more accessible and safe for you. Pay attention to lighting, area rugs, slippery floors and anything you think may be unsafe or hard to do with functional aids.

12. Practice walking with crutches and the range of motion exercises

After your joint replacement operation, you will need crutches for walking. Practice using them before your surgery. You can collect a pair of crutches from the medical aid service at your health centre. Borrowing the equipment is free of charge, and you don't need a referral to do that.

Don't forget to do your range of motion exercises, as they will maintain both motion and muscle tone.

See the exercise instructions on pages 13–15.

Visiting the surgery outpatient clinic

Your health centre has referred you to blood tests in order to track your health, but you may need to have some more specific tests or X-ray images taken before your surgery. Make sure to go to all tests you are referred to. You will receive instructions on them by mail.

Before your first visit to the surgery outpatient clinic, you will receive an electronic Omavointi questionnaire concerning your health and ability to function. Use electronic identification (such as online banking codes) to log into the questionnaire.

Remember to bring an updated list of your current medication with you.

An orthopaedist at the outpatient clinic will talk about the date or possible postponing of your surgery with you, and you will also see a nurse and a physiotherapist. During your visit, you will receive the date for your surgery, instructions on how to prepare for it, and a date for a joint replacement training.

If you wish so, it's possible to go home from the hospital on the day of your surgery. During your visit to the outpatient clinic, your doctor will discuss whether this is a possibility for you.

Returning to work

After a joint replacement operation, the hospital will write you a certificate for a 4-week sick leave. If you are entitled to occupational health services, your plan for returning to work will be made there. Otherwise, the plan will be made in your own health centre before the end of your 4-week sick leave. Please book a time for this work ability assessment from your occupational health provider or health centre as soon as you know the date of your operation.

Change in health after your visit

Please contact the hospital if you suspect you may have an illness, infection, skin cut or any other change in your health that could prevent the surgery (for example cough, runny nose, fever or stomach flu). Remember to contact the hospital also if there are any changes to your medication.

Preparing for your surgery

Take a shower the night before your surgery. Make sure to remove all jewellery, piercings and nail polish before you shower. By washing yourself thoroughly, you decrease the risk of infections. Pay close attention to armpits, groin area, genitals, skin folds, and belly button. Use a clean towel to dry yourself, and make sure you have fresh sheets in your bed. Do not shave for 7 days before your surgery. Do not moisturise the skin in the surgery area after you shower.

The night before your surgery, you can eat and drink normally **until 24:00**. After that, **you are not allowed to eat or drink anything before your surgery**.

When packing for hospital, take only what you need with you. If you wish, you can bring your own shoes to increase safety.
Do not bring jewellery or large amounts of cash with you.

The hospital is not responsible for your lost or broken belongings.

Bring only the essentials with you

- The invitation letter and your Kela card or identity card
- Of your medication: Dinit spray, eye drops, insulin pens, asthma inhalers
- Mobile phone and charger
- Crutches and other aids you may use: hearing aid, eyeglasses, CPAP
- Personal hygiene products
- This guidebook

On the day of your surgery

1. Morning at home

Take your personal medication according to your doctor's advice (see the end of this guidebook) with a small amount of water (1 dl). Do not eat or drink anything else. You can brush your teeth.

On the day of your surgery, don't use lotion in the area of the surgery, and make sure you are not wearing any makeup. Please don't wear any fragrances either. Dress in fresh clothes, and make sure they are as practical as possible for when you go home after the surgery.

2. Arriving at the hospital

When you arrive, use the Nova 1 entrance. Use the G block elevator and go to the 3rd floor. Use the self-check-in kiosks to let us know that you have arrived. After the check-in, our staff will take you to a dressing room to change into your surgery clothes. Our staff will assist you, if needed.

A nurse will tell you about how the day will proceed, and make sure that you have followed all the pre-surgery instructions, that your skin is not broken, and that you haven't been eating or drinking anything. You will receive preoperative medication to ease the pain after surgery, help with anxiety, and prevent nausea.

You may need to wait for your surgery. While you are waiting, you can watch tv, read magazines or use your own mobile devices in the lobby.

Use the bathroom before you are taken to the operation. Make sure to pay attention to hand hygiene and use disinfectant on your hands.

3. Anaesthesia and medication

Most joint replacement operations are performed under spinal anaesthesia. Your legs will go numb from waist down for about 3–4 hours. In addition to anaesthesia, you can ask for sleeping medication so that the noise from the surgery won't disturb you. All joint replacement patients need to take antibiotics prior to the surgery to prevent infections.

After the surgery

After the surgery, you will be taken into a recovery room where your progress can be monitored to make sure that you are feeling well, the anaesthesia wears out, and your pain medication is sufficient. After this, you will be taken either to the discharge unit or to a ward. The wards are located on the 5th floor of blocks C and J. The visiting hours for the wards are 12:00–20:00.

1. Rehabilitation starts on the day of surgery

As soon as the anaesthesia wears out, you should:

- move the operated joint
- actively straighten and bend your ankles
- activate and then relax the muscles of your thighs and buttocks

During the first 6 hours after your surgery, a nurse or a physiotherapist will help you get out of bed. You are free to walk, but use crutches until you can walk as usual.

Tell the staff if you’re not feeling well or you need help with pain management. Take the pain medication as prescribed. However, at this stage it’s impossible to get rid of all pain. You can speed up the recovery process by being active and doing things independently. Remember to start doing the range of motion exercises.

Walking with crutches

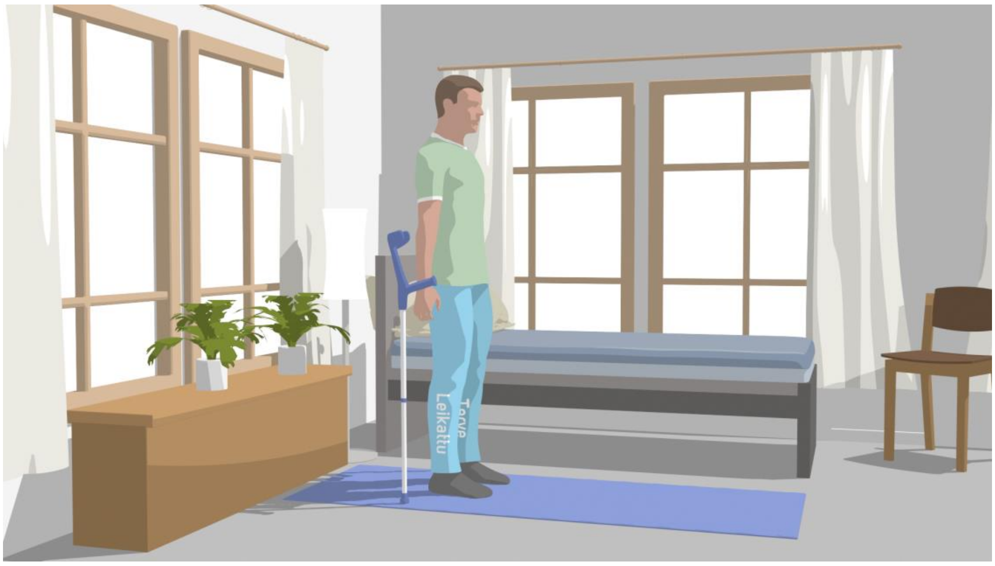

Adjusting the height. Stand with your hands by your side. The height is right when the handle is on level with your wrist.

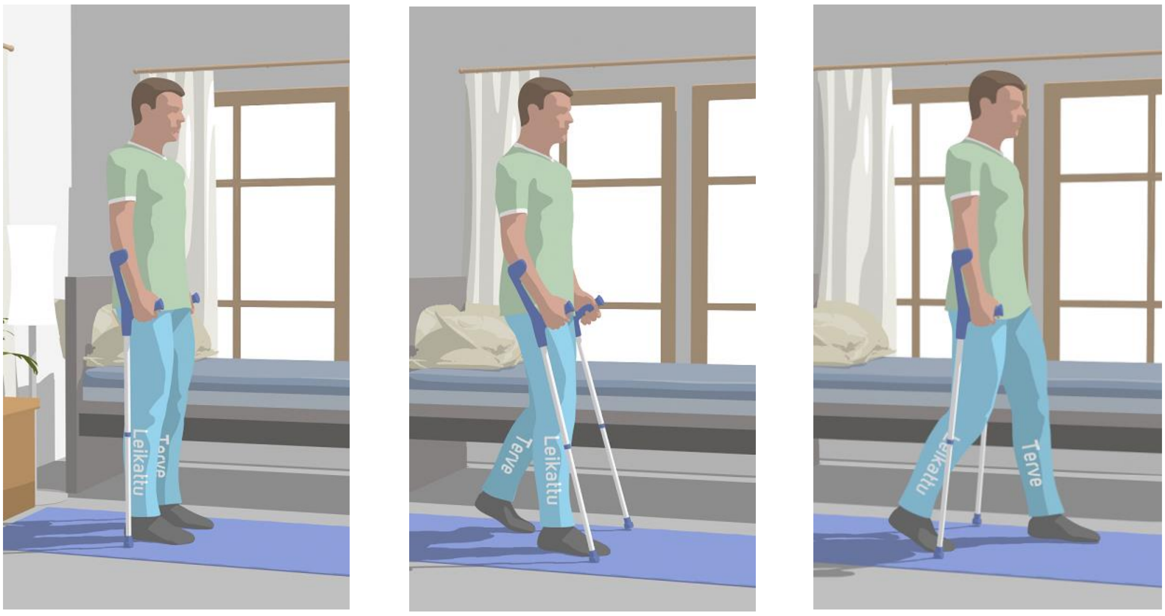

Walking with crutches on even ground. Take the crutches and your operated leg forward. With your good leg, take a step past the operated leg. The steps should be close in length.

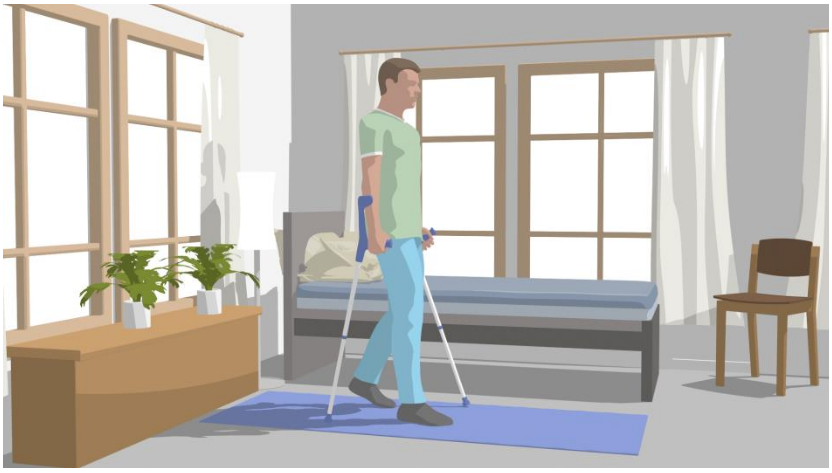

Walking with crutches at alternate pace. Take the opposing hand and leg forward at the same time.

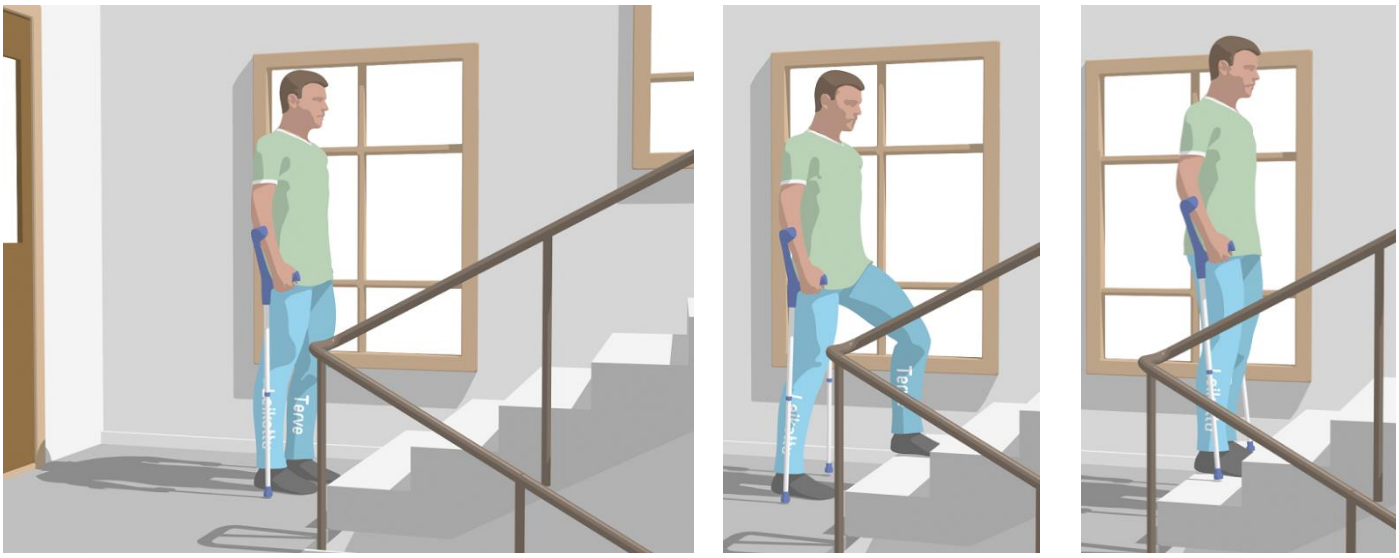

Climbing stairs. Raise your good leg on the next step. With the weight on the good leg, take your operated leg and the crutches on that same step.

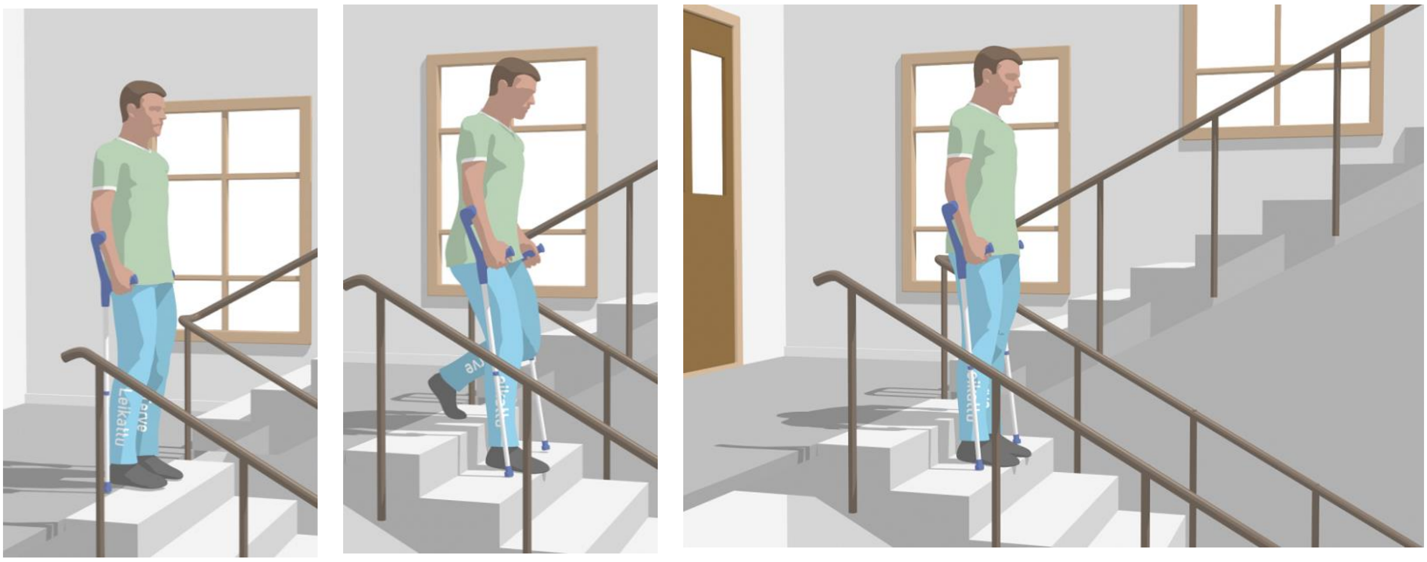

Descending. Take your operated leg and crutches on the next step. Then take the good leg on the same step. You can use hand rails to help you.

Knee exercises

Here are five exercises to enhance the range of motion and the strength of your knee. Go through them on a regular basis before your surgery, and keep practicing for six weeks after the surgery to restore the range of motion. After four weeks from your surgery, you should be able to both straighten your knee and have it bended 90 degrees. If the range of motion has not improved as planned, please contact a physiotherapist.

Tips on exercise

- If necessary, take pain medication 30 minutes before exercising.
- Practice two times a day.
- Repeat each exercise 3–10 times.
- Go through the routine 1–3 times per session.
- Start with three repeats per exercise and go through the routine once.
- As you make progress, start adding repeats to exercises and routines.

Exercise 1

Strengthen your thighs

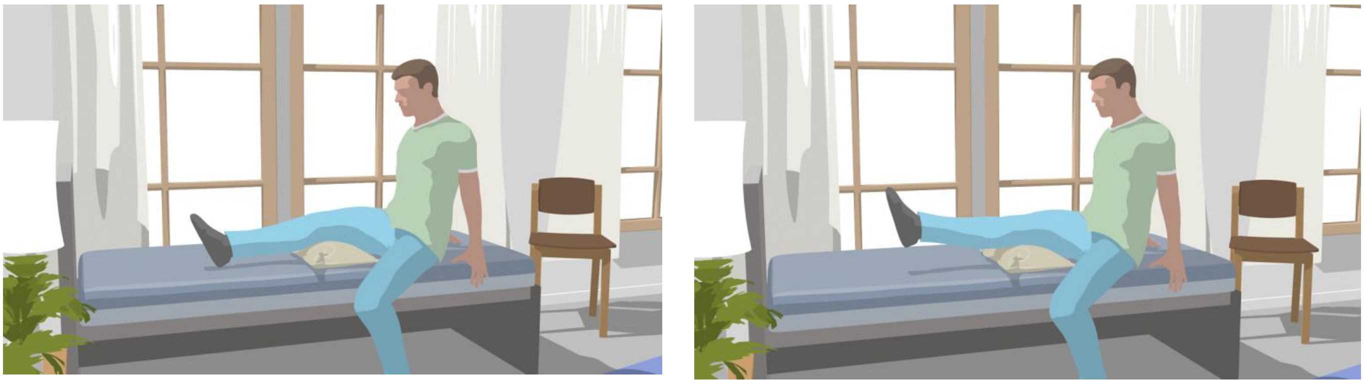

Activate the muscles of your thigh. Straighten your knee as far as you can, and hold it there for 5 seconds. Lower your leg carefully and relax it. Practice also without a pillow, using only your thigh muscles.

Exercise 2

Practice range of motion

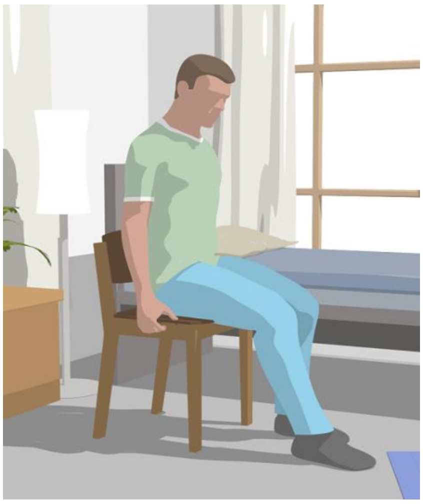

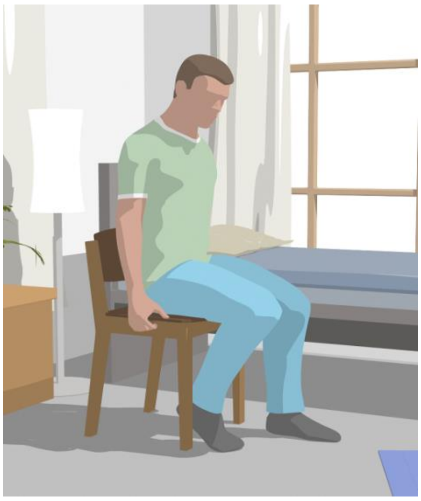

Bend your knee as far as you can, gliding your soles and toes on the floor. Your heels can rise from the floor. Hold the stretch for 15–30 seconds.

Exercise 3

Strengthen your thighs

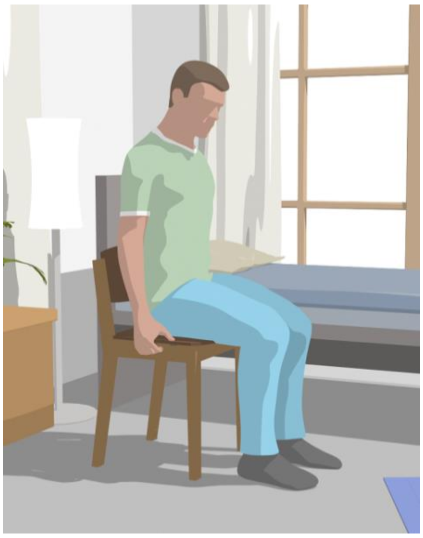

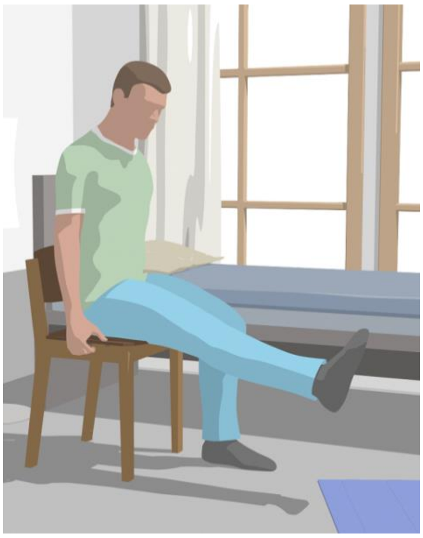

Straighten your knee as far as you can. Lower your leg carefully.

Exercise 4

Practice range of motion

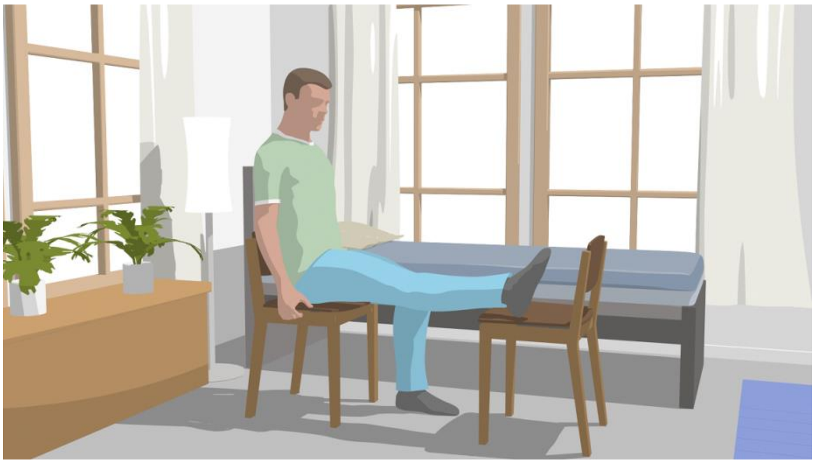

Sit on a chair and place your heel on another chair. Let your knee straighten, and hold the stretch for 20–30 seconds. Make sure that your kneecap faces straight up towards the ceiling at all times. Repeat several times throughout the day.

Exercise 5

Practice walking

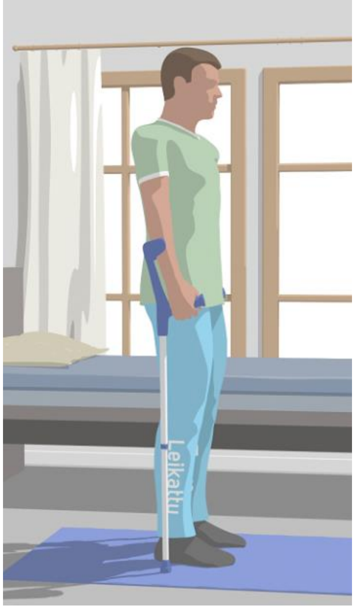

A

Stand with your feet hip-width apart, supported by crutches.

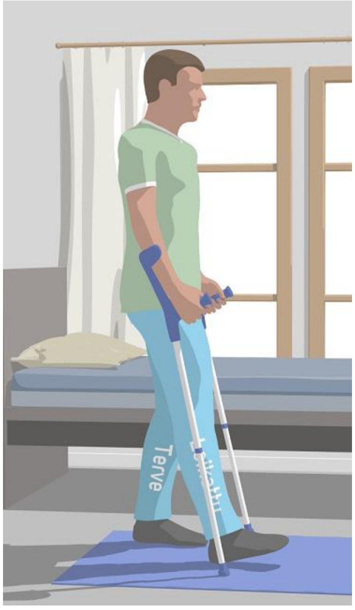

B

With your good leg, take a step to the back and put the weight on that leg. This raises the toes of your operated leg, and only the heel will stay on the ground.

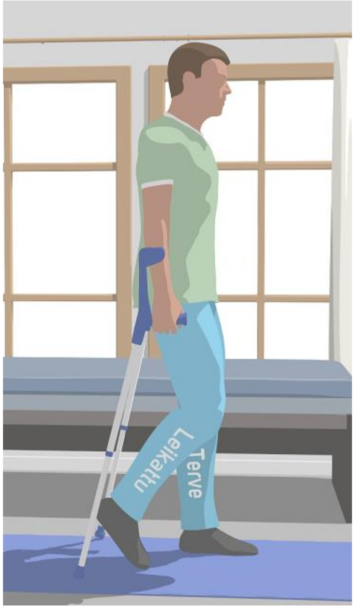

C

Now shift the weight to your operated leg. Swing the good leg forward so that the weight is on the operated leg, and the heel of the operated leg is raised form the ground.

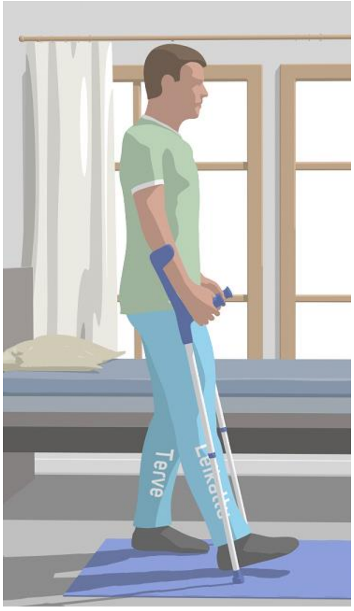

D

Shift the weight back to your good leg, and swing it to the back. The weight is now on the good leg, and the toes of your operated leg are raised. Stop when your good leg is back, and repeat the back and forth -steps. Repeat the steps with your other leg, as well.

2. Preventing thrombosis

To prevent thrombosis, it is important that you are as active as possible and get on your feet several times a day. After the surgery, your doctor will assess whether you also need medication to prevent thrombosis.

3. Going home

The hospital staff will assess your ability to function. You can leave the hospital after you meet all the preconditions that have been agreed on. For your safety, we wish your support person comes to listen the instructions given by your doctor and nurse. You will get all necessary prescriptions, sick leave forms, an infection awareness form, and the date for your post-surgery check-up (the last may also be sent to you). Keep in mind that patients can be discharged from the hospital in the evening, as well. You won't necessarily meet your surgeon before you leave the hospital.

When can I go home?

You can leave the hospital when you meet all these preconditions:

- You can dress yourself
- You can get out of bed and stand up from a chair by yourself
- You can take care of your personal hygiene
- You can walk 40 meters, and if necessary, climb stairs
- You have someone who can help you at home

After day-surgery, you also need to:

- have someone to take you home, and come listen to the instructions given by your doctor and nurse
- have someone to stay at home with you
- have had the surgery under local anaesthesia
- live close to the hospital (approximately 30 mins)
- feel well enough
- manage with your pain

At home after surgery

1. Pay attention to the wound

The wound is closed using metal staples, stitches or soluble stitches, and covered with either bandage or breathable tape.

You may notice bruising and swelling around the wound or in larger areas, but they will get better gradually. Swelling may occur for weeks, but some experience it even after a few months. You can try keeping your leg elevated and using cold compressions to ease the swelling.

It is normal for the wound to feel warm for a few months. A slight change in your temperature is a normal part of the healing process, so you don't need to worry about that.

After the surgery, your haemoglobin levels are likely to drop, but they will restore gradually.

The need for pain medication varies greatly, but for most patients it is needed for the first few weeks or months.

Make sure the wound heals

- Keep the wound dry for the first 24 hours after the surgery. After this, you can go to shower as usual.
- Pat the wound dry (you don't have to change the bandage after taking a shower).
- If the bandage is dirty or covered in discharge, change it.
- When you no longer notice discharge, you can start using surgical tape to protect the wound.
- You can go to sauna 24 hours after the removal of either stitches or staples, and you no longer notice any discharge.

Contact the hospital in case you notice:

- bleeding you cannot stop
- the wound opening
- discharge that smells and comes through the bandage, or your temperature is over 38 °C

Contact a health centre in case you notice:

- warmth, swelling or redness that is unlike the situation after your surgery
- pain that gets worse or won't go away

Removal of the staples or stitches

When you're discharged from the hospital, you doctor will tell you when the staples or stitches can be removed. Book a removal appointment with a nurse at your own health centre. Soluble stitches don't need to be removed.

2. Pain management after surgery

Take the pain medication prescribed to you according to the instructions. Exercise, cold compressions and elevating your leg may lessen the need for medication. Pain should not keep you from sleeping, moving or exercising. The need for medication varies from patient to patient, but you should be able to take less and less painkillers and eventually stop taking them as time goes by. If you have taken pain medication according to the instructions but the pain doesn't stop, call the number at the back of this guidebook.

3. A call from a nurse

If you go home on the day of your surgery, you will receive a phone call from a nurse the next day. You will be called at 12:00–15:00, as it is important to check that your recovery process has started well.

4. Recovery process at home

You should be able to go back to your normal day-to-day life gradually. The length of the recovery process varies greatly, but it may take several months or even a year.

You can ease the process by

- staying active
- taking care of your daily routines, and
- practicing the range of motion regularly

During the first few weeks, you should avoid activities that include heavy straining, but it is up to you to assess the straining level. The recovery process cannot move on without enough activity and rest, so try to find a balance between them. Remember to take breaks in between chores, and start with short walks several times a day. Take care of your daily routines little by little, and rest between them.

After your wound is closed, you can start low-intensity training in water or on an exercise bike. After six weeks from your surgery, you can start exercising muscle strength. Start with low-intensity straining, and raise the level of straining gradually. You can contact a physiotherapist at your health centre in case you need help with exercise-related matters.

You can start driving a car after you no longer need aids to move around or take strong painkillers (marked with red warning triangles). As a passenger, you can travel by car as soon as you're discharged from the hospital.

There are no restrictions to sexual activity.

Living with joint replacement

1. Avoid infecting your joint replacement

To avoid infecting your joint replacement, make sure to:

- keep your long-time diseases in control
- treat all infections and ulcers properly
- take good care of oral health
- tell your dentist about your joint replacement

If you think your joint replacement might be infected, contact your health centre.

2. Exercise

Take care of your health by training your endurance, muscle strength, balance and mobility on a regular basis. You can go back to your previous hobbies.

Make sure to get at least 2.5 hours of moderate or 1.25 hours of vigorous endurance training a week. Divide it throughout the week. Good forms of endurance training include:

- walking and Nordic walking
- biking outdoors or on an exercise bike
- aquatic sports
- skiing
- dancing
- group exercise
- outdoor activities such as fishing, hunting and berry-picking
- housework and gardening

Practice your strength, balance and mobility at least two times a week. Good forms of exercise include:

- going to the gym
- gymnastics at home
- aquatic sports
- group exercise
- dancing
- stretching
- walking on uneven ground

Keep in mind that high-impact sports and overweight may shorten the life span of your joint replacement.

3. Travelling by air

If you face any troubles during airport security checks, tell the staff about your joint replacement. You won’t need a certificate, all you need to do is inform the staff.

Checklist before surgery

- [ ] Make plans with your friends and family
- [ ] Take care of your long-time diseases
- [ ] Check your skin condition
- [ ] Visit a dentist
- [ ] Take care of nutrition
- [ ] Two months before surgery, quit:
    - smoking
    - alcohol
    - omega-3 and other natural health supplements
- [ ] Collect crutches
    - practice walking with crutches
- [ ] Prepare your home
    - ask for help and book it, if necessary
    - make sure your home is safe and accessible
- [ ] Take care of all medical examinations you’re referred to
- [ ] Fill in the Omavointi-questionnaire
- [ ] Attend the joint replacement training
- [ ] Study the preconditions for going home after the surgery

Journey to the hospital

- If you are eligible for Kela taxi reimbursement, remember to book your taxi through the central dispatch number 0100 87650 no later than 14:00 the day before your surgery.

Notes and your own questions

Important information on your surgery

Day of surgery:	Arrive by:
Medication you should stop taking before surgery:	
Medication on the day of surgery:	
Further examinations:	
Removal of stitches or staples:	

Contact information

In case of need, please contact:

- Inquiries on the surgery, changes in your health:
  014 269 5205, Mon–Fri at 9:00–11:00 and 13:00–14:00
- Sudden cancellations on the day of your surgery:
  014 269 5959, Mon–Fri at 7:00–9:00
- Questions on the surgery or recovery process (nurse):
  014 269 1220, Mon–Fri at 9:00–11:00, weekends and public holidays 014 269 1036.
- Social worker at the surgery outpatient clinic (financial assistance):
  014 269 1555, Mon–Fri at 8:00–16:00
- Physiotherapist at the surgery outpatient clinic:
  014 269 5105, Mon–Fri at 12:00–14:00.

For more information on joint replacement operations, visit our website www.sairaalanova.fi/tekonivelleikkaus, or www.nivel.fi (in Finnish).

Omavointi questionnaire: www.omavointi.fi/ksshp/ovui/#/login

All your health records are available from the My Kanta service: www.kanta.fi/en/omakanta.
