# Supplementary material for: Effectiveness of Gamification in Knee Replacement Rehabilitation: Protocol for a Randomized Controlled Trial With a Qualitative Approach
Source: JMIR Res Protoc. 2022 Nov 28;11(11):e38434. doi: 10.2196/38434 (PMC9745648; doi:10.2196/38434)
Supplement: Multimedia Appendix 4 [file resprot_v11i11e38434_app4.pdf]

Multimedia Appendix 4: Laddering interview stimuli

Post-operative rehabilitation is recommended to continue at home after patients having the operation of total knee arthroplasty. Think about your post-operative rehabilitation process at home, which two of the following would interest you? You can also invent your own. Please do not limit yourself to the examples described in the cases.

1. During the process of the post-operative rehabilitation, it is important to do the home exercise appropriately every day as expected. Of course, some people may find it hard or boring to repeat the exercise and may quit sometimes. Did you do the rehabilitation exercise everyday as expected? (Why?)

2. During the process of the post-operative rehabilitation, you may need some resources to complete the exercise. For example, you may need access to information of how to do the exercise, with certain devices, and the knowledge to do it appropriately and so on. Except for the available resources, you can also think about resources that you need but not available. During this process, what is meaningful to you? (Why?)

3. During the process of the post-operative rehabilitation, you may need some interactions with others. For example, you may need to connect with hospital staffs through certain channels such as phone call or email. It can also happen that you could not find any way to connect with them when it is needed. The communication process may go on well or wrong. You may always trust others during the interaction or not. During this process, what is meaningful to you? (Why?)

4. During the process of the post-operative rehabilitation, some people may feel satisfied, for example, because they are recovering little by little. While some people may feel dissatisfied for example, because they went through some problems, or the rehabilitation is not going on well as expected. Are you satisfied or dissatisfied with the post-operative rehabilitation? (Why?)
