# Supplementary material for: Effectiveness of Gamification in Knee Replacement Rehabilitation: Protocol for a Randomized Controlled Trial With a Qualitative Approach
Source: JMIR Res Protoc. 2022 Nov 28;11(11):e38434. doi: 10.2196/38434 (PMC9745648; doi:10.2196/38434)
Supplement: Multimedia Appendix 6 [file resprot_v11i11e38434_app6.docx]

# Multimedia Appendix 6: CONSERVE-SPIRIT Extension for BEE-RCT

| **CONSERVE-SPIRIT Extension: [18^th^ October 2021]** | | | | | | |
| --- | --- | --- | --- | --- | --- | --- |
| **Item** | **Item Title** | **Description** | | | **Page No.** |  |
| I. | Extenuating Circumstances | Describe the circumstances and how they constitute  extenuating circumstances.  COVID-19 and its effect on elective TKR surgeries, societal lockdowns, and increased fear of exposing to the disease. This caused 1) a temporary discontinuation and unremitting slowdown in recruitment and 2) interruptions to data collection. | | | 11, 12, 15 |  |
| II. | Important Modifications | a. Describe how the modifications are important  modifications.  Modifications were done to minimize the missing data and its’ effect on study’s ability to address effectiveness and statistical power. | | | 9 |  |
|  |  | b. Describe the impacts and mitigating strategies,  including their rationale and implications for the  trial.   - revised outcome collection procedures - revised analysis plan | | | 9, 11, 12 |  |
|  |  | c. Provide a modification timeline.  Timeline for modifications was year 2020 to 2021. | | | 14 |  |
| III. | Responsible Parties | State who planned, reviewed and approved the  modifications.  Research group planned and implemented the modifications that were approved by principal investigators. | | | 9 |  |
| IV: | Interim data | If modifications were informed by trial data, describe how the interim data were used, including whether they were examined by study group, and whether the individuals reviewing the data were blinded to the treatment allocation | | | n.a. |  |
| **SPIRIT Item and Number** | | **No Change** | **Impact*** | **Mitigating**  **Strategy**** | **Page No.** |  |
| 1 | Title | x |  |  |  |  |
| 2 | Trial registration | x |  |  |  |  |
| 3 | Protocol version | x |  |  |  |  |
| 4 | Funding | x |  |  |  |  |
| 5 | Roles and responsibilities | x |  |  |  |  |
| 6 | Background and rationale | x |  |  |  |  |
| 7 | Objectives | x |  |  |  |  |
| 8 | Trial design | x |  |  |  |  |
| 9 | Study setting | x |  |  |  |  |
| 10 | Eligibility criteria | x |  |  |  |  |
| 11 | Interventions | x |  |  |  |  |
| 12 | Outcomes | x |  |  |  |  |
| 13 | Participant timeline | x |  |  |  |  |
| 14 | Sample size |  | x |  | 11, 12, 14 |  |
| 15 | Recruitment |  | x |  | 10 |  |
| 16 | Allocation | x |  |  |  |  |
| 17 | Blinding (masking) | x |  |  |  |  |
| 18 | Data collection methods |  | x | x | 8, 10 |  |
| 19 | Data management | x |  |  |  |  |
| 20 | Statistical methods |  | x | x | 12 |  |
| 21 | Data monitoring | x |  |  |  |  |
| 22 | Harms | x |  |  |  |  |
| 23 | Auditing | x |  |  |  |  |
| 24 | Research ethics approval | x |  |  |  |  |
| 25 | Protocol amendments | x |  |  |  |  |
| 26 | Consent or assent | x |  |  |  |  |
| 27 | Confidentiality | x |  |  |  |  |
| 28 | Declaration of interests | x |  |  |  |  |
| 29 | Access to data | x |  |  |  |  |
| 30 | Ancillary and post-trial care | x |  |  |  |  |
| 31 | Dissemination policy | x |  |  |  |  |
| 32 | Informed consent materials | x |  |  |  |  |
| 33 | Biological specimens | x |  |  |  |  |
| *Aspects of the trial that are directly affected or changed by the extenuating circumstance and are not  under the control of investigators, sponsor or funder.  **Aspects of the trial that are modified by the study investigators, sponsor or funder to respond to the  extenuating circumstance or manage the direct impacts on the trial.  The CONSERVE-SPIRIT Checklist is licensed by the CONSERVE Group under the Creative Commons  Attribution-NonCommercial-NoDerivs 4.0 International license. | | | | | |  |
